# Projecting coral responses to intensifying marine heatwaves under ocean acidification

**DOI:** 10.1111/gcb.15818

**Published:** 2021-08-29

**Authors:** Shannon G. Klein, Nathan R. Geraldi, Andrea Anton, Sebastian Schmidt‐Roach, Maren Ziegler, Maha J. Cziesielski, Cecilia Martin, Nils Rädecker, Thomas L. Frölicher, Peter J. Mumby, John M. Pandolfi, David J. Suggett, Christian R. Voolstra, Manuel Aranda, Carlos. M. Duarte

**Affiliations:** ^1^ Red Sea Research Center (RSRC) and Computational Bioscience Research Center (CBRC) King Abdullah University of Science and Technology (KAUST) Thuwal Kingdom of Saudi Arabia; ^2^ Red Sea Research Center (RSRC) King Abdullah University of Science and Technology (KAUST) Thuwal Kingdom of Saudi Arabia; ^3^ Department of Animal Ecology & Systematics Justus Liebig University Giessen Germany; ^4^ Climate and Environmental Physics Physics Institute University of Bern Bern Switzerland; ^5^ Oeschger Centre for Climate Change Research University of Bern Bern Switzerland; ^6^ Marine Spatial Ecology Lab School of Biological Sciences The University of Queensland Brisbane Queensland Australia; ^7^ Australian Research Council Centre of Excellence for Coral Reef Studies School of Biological Sciences The University of Queensland Brisbane Queensland Australia; ^8^ Climate Change Cluster Faculty of Science University of Technology Sydney Sydney New South Wales Australia; ^9^ Department of Biology University of Konstanz Konstanz Germany

**Keywords:** climate change, coral bleaching, endosymbiosis, greenhouse gas emissions scenario, ocean warming

## Abstract

Over this century, coral reefs will run the gauntlet of climate change, as marine heatwaves (MHWs) become more intense and frequent, and ocean acidification (OA) progresses. However, we still lack a quantitative assessment of how, and to what degree, OA will moderate the responses of corals to MHWs as they intensify throughout this century. Here, we first projected future MHW intensities for tropical regions under three future greenhouse gas emissions scenario (representative concentration pathways, RCP2.6, RCP4.5 and RCP8.5) for the near‐term (2021–2040), mid‐century (2041–2060) and late‐century (2081–2100). We then combined these MHW intensity projections with a global data set of 1,788 experiments to assess coral attribute performance and survival under the three emissions scenarios for the near‐term, mid‐century and late‐century in the presence and absence of OA. Although warming and OA had predominately additive impacts on the coral responses, the contribution of OA in affecting most coral attributes was minor relative to the dominant role of intensifying MHWs. However, the addition of OA led to greater decreases in photosynthesis and survival under intermediate and unrestricted emissions scenario for the mid‐ and late‐century than if intensifying MHWs were considered as the only driver. These results show that role of OA in modulating coral responses to intensifying MHWs depended on the focal coral attribute and extremity of the scenario examined. Specifically, intensifying MHWs and OA will cause increasing instances of coral bleaching and substantial declines in coral productivity, calcification and survival within the next two decades under the low and intermediate emissions scenario. These projections suggest that corals must rapidly adapt or acclimatize to projected ocean conditions to persist, which is far more likely under a low emissions scenario and with increasing efforts to manage reefs to enhance resilience.

## Graphical abstract

Over this century, coral reefs will run the gauntlet of climate change, as marine heatwaves (MHWs) become more intense and frequent, and ocean acidification progresses. Here, we projected future MHW intensities for tropical regions and then parametrized a global data set of coral experiments. Our analyses project increasing instances of coral bleaching and substantial declines in coral productivity, calcification and survival within the next two decades under the low and intermediate emissions scenario.

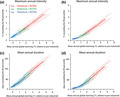

## 1 INTRODUCTION

Marine heatwaves (MHWs, Hobday et al., 2016) are distinct events of extreme sea temperatures lasting days to months and projected to intensify with anthropogenic climate change (Collins et al., 2019; Frölicher et al., 2018; Oliver et al., 2018), with potentially far‐reaching implications for marine ecosystems (Smale et al., 2019). Intense MHWs have already caused severe impacts on many critical habitats over the past two decades, including seagrass meadows (Marbà & Duarte, 2010; Thomson et al., 2015), kelp forests (Wernberg et al., 2018) and coral reefs (Hughes, Anderson, et al., 2018). Coral reefs have suffered coherent, global‐scale impacts across their range (Hughes, Kerry, et al., 2017) and are likely one of the most vulnerable ecosystems to the contemporary threat of intensifying MHWs (Bindoff et al., 2019). Thermal stress during prolonged MHWs triggers the collapse of the relationship between corals and their symbiotic microalgae, causing corals to appear colourless or ‘bleached’, often resulting in widespread coral death (Baker et al., 2008; Spalding & Brown, 2015). Over the past three decades, large‐scale bleaching events have become increasingly common on reef ecosystems across the tropics (1997–1998, 2008–2010 and 2014–2017), and strong evidence links these phenomena directly to severe MHWs (Eakin et al., 2016; Heron et al., 2016; Hughes, Anderson, et al., 2018; Hughes, Kerry, et al., 2018).

Accelerated efforts towards conserving coral reef ecosystems in the Anthropocene era call for a clear understanding of the numerous drivers that will harm coral reefs (Hughes, Barnes, et al., 2017). The latest and most inclusive assessments of coral reef susceptibility to climate change, conducted by the Intergovernmental Panel on Climate Change (IPCC) (Hoegh‐Guldberg et al., 2018; Bindoff et al., 2019), recognize MHWs as a primary threat to reef ecosystems and acknowledge that ocean acidification (OA, caused by projected increases in ocean CO_2_ levels) will place reefs under greater risk. These IPCC assessments concluded that the number of coral reefs will decline by a further 70%–90% at 1.5°C global warming (above pre‐industrial levels) and decline by 99% at 2°C assessed via modelled temperature simulations (Frieler et al., 2013; Schleussner et al., 2016). These simulations assume that higher CO_2_ levels will reduce coral tolerance to heat stress based on the findings of a single published experiment testing two coral species (Anthony et al., 2008). However, the generality that all coral taxa are more vulnerable to heat stress at higher CO_2_ levels is yet to be tested. Indeed, we still lack a comprehensive understanding of how, and to what degree, OA will affect the resilience of corals to intensifying MHWs.

Here, we first modelled future MHW intensities for tropical and subtropical regions according to three global warming scenarios. We then synthesize the results of coral experiments conducted across the globe to project changes in coral performance and survival under future MHW scenarios throughout this century and assess the additional influence of OA. Specifically, we compiled a data set of 1,788 independent experimental measures assessing coral responses to warming and increased CO_2_ levels conducted in tropical and subtropical regions from 172 published studies of 87 widely distributed coral species (Figures S2–S5; Table S2). We then combined experimental results of coral performance as temperature and CO_2_ levels increase with our MHW intensity projections to deliver estimates of coral performance and survival under three representative concentration pathway (RCP) scenarios for the near‐term (2021–2040), mid‐century (2041–2060) and late‐century (2081–2100) in the presence and absence of OA. Although MHWs and OA are unavoidably linked owing to anthropogenic CO_2_ emissions (Pachauri et al., 2014), we also evaluated coral performance in the absence of OA to determine how corals would respond to warming if temperature was the sole stressor.

Reef‐building corals are not singular entities, but instead comprise a dynamic association between the coral host, micro‐algal symbionts and a range of bacteria, fungi and viruses (Rohwer et al., 2002)—termed the ‘coral holobiont’. To provide insight into the resilience of corals to future MHWs, we used experimental measurements of physiological performance and conditions, which included the eight most commonly measured holobiont attributes in the reviewed studies: symbiont density, chlorophyll *a* (chl*a*) content, photochemical efficiency, photosynthesis, respiration, calcification, growth and survival (Table S3). We used effect sizes determined from experimental results as the foundation to project the effect of warming and OA on these coral attributes.

## 2 MATERIALS AND METHODS

### 2.1 Projections of MHWs under global warming scenarios

We used the same daily sea surface temperature (SST) and surface atmospheric temperature data as used in the study by Frölicher et al. (2018), but extended the analysis with an additional future greenhouse gas emissions scenario (RCP4.5). In particular, we used output from 12 coupled Earth system models that participated in the fifth phase of the Coupled Model Intercomparison Project (CMIP5): CanESM2, CSIRO‐Mk3‐6‐0, GFDL‐CM3, GFDL‐ESM2G, GFDL‐ESM2M, HadGEM2‐ES, IPSL‐CM5A‐LR, IPSL‐CM5A‐MR, MIROC‐ESM, MPI‐ESM‐LR, MPI‐ESM‐MR and MRI‐CGCM3. All simulations were run over the historical 1861–2005 period and follow the RCP scenarios RCP2.6, RCP4.5 and RCP8.5 over 2006–2100. We defined a MHW when the daily SST exceed either the 90th (a ten‐in‐a‐hundred‐days event) or the 99th (a one‐in‐a‐hundred‐days event) percentile, calculated from multi‐centennial pre‐industrial control simulations (sensu, Frölicher et al., 2018; Hobday et al., 2016; Oliver et al., 2018). For each MHW, we calculated the duration (number of days of percentile threshold exceedance) and maximum intensity (maximum SST anomaly with respect to the percentile threshold over the duration of the heatwaves). We then calculated annual statistics. The analysis simulated changes in MHWs for tropical and subtropical regions (between +30 and −30 degrees latitude), covering >95% of the geographical locations where our experimental data were obtained (Figure S3).

### 2.2 Literature search

The published experimental literature on responses of calcifying corals (Scleractinia: Anthozoa) to warming and elevated *p*CO_2_ in single and combined treatments was searched using the Web of Science® database. The original search collected data for all benthic cnidarians, but due to low replication of observations for non‐calcifying taxa, we report only data for calcifying corals. The search was done on the 14 September 2017 and produced 1059 papers using the following search term: (coral OR octocoral OR anemone OR cassiopea OR scleractinia* OR corallimorpharia* OR gorgonia*) near/10 (impact OR effect OR response OR affect OR stress*) near/10 (temperature OR warming OR heat OR thermal OR “climate change” OR acidification OR *CO_2_ OR pH OR hypercapnia OR acidosis) NOT fish (Figure S2).

### 2.3 Study selection criteria

Each publication was assessed for suitability, and we initially retained those that: (1) assessed responses of benthic cnidarian taxa, (2) reported empirical measures of biological responses of benthic cnidarians to warming or elevated *p*CO_2_ in single and in combined treatments relative to those of control (ambient) conditions and (3) reported mean values, sample sizes (*n*) and a measure of variance around the mean (e.g. SE, SD and CI) for the biological responses under both control and treatment conditions (Figure S2). We used the same control (ambient) conditions as defined by the authors of the published studies. Published literature used in the analysis was not limited to the studies of manipulative laboratory experiments, but included empirical observations from ‘model’ ecosystems (e.g. CO_2_ vent sites). However, data obtained from observations of ‘model’ ecosystems were extracted only if both control and treatment sites were included and the study included efforts to control other, potentially confounding variables among sites. A small number of published studies of OA reported only pH levels and did not characterize *p*CO_2_ levels of the treatments tested. We thus restricted the data set to experimental assessments that verified and reported the full carbonate system to estimate *p*CO_2_ levels in the treatments tested. Laboratory experiments that reduced pH using acid–base manipulation (i.e. manipulated total alkalinity rather than dissolved inorganic carbon) were excluded from the data set because this method does not accurately replicate changes to ocean carbonate chemistry (Gattuso & Lavigne, 2009).

### 2.4 Data extraction and data set characteristics

To assess the various biological responses of calcifying corals to warming, elevated *p*CO_2_ and the stressors combined, the following were selected as response attributes: symbiont density, chl*a* content, photosynthesis, photochemical efficiency (YII), calcification, host growth, host survival and dark (holobiont) respiration. Mean responses, sample size (*n*) and measures of variance (e.g. SE, SD and CI) were extracted for biological responses measured under ambient (control) and manipulated conditions. Data extraction from figures was done using the image analysis software Graph Click© (for Mac OS, version 3.0) and Web Plot Digitizer © (for PC, version 4.0). In cases where multiple levels within a single factor were examined within a study, levels were entered as independent experiments. If a study measured biological responses of multiple species or life‐history stages, these data were also included as individual experiments. Likewise, if a study measured more than one of the specified response variables, all responses were included in the analyses. However, if a study recorded multiple metrics considered to reflect a single coral process, then only the most ‘inclusive’ variable was included (sensu, Kroeker et al., 2010, 2013).

Due to low replication for non‐calcifying taxa, 32 studies of non‐calcifying taxa were excluded from the data set (Figure S2). We further restricted the data set to experimental assessments conducted in tropical and subtropical regions (between +35 and −35 degrees of latitude) and excluded 11 studies that assessed the responses of deep‐sea corals to ensure the relevance of surface MHW scenarios (Figure S2). We excluded eight studies that assessed the responses of calcifying corals that did not measure any of the selected eight responses and excluded experimental scenarios of acidification that increased *p*CO_2_ levels by more than 800 µatm. The resulting data set comprised 1,788 replicated experiments from 172 published studies (Table S5) of 87 reef‐building coral species (Table S2). The experiments tested temperatures spanning 25 and 34°C (and warming scenarios of +0.5 and +13°C), and *p*CO_2_ levels spanning from 475 to 1200 µatm (and elevated CO_2_ scenarios between +100 and +800 µatm), relative to control values of 24–30°C and 297–486 µatm respectively (Figure S5). Since our effect size calculations are standardized for differences in temperature and *p*CO_2_ levels between the control and experimental treatments, we were able to include even pessimistic scenarios, which are often excluded from meta‐analyses. The overall mean duration of the experimental assessments was 22.2 days.

### 2.5 Response metrics

We quantified the temperature dependence or sensitivity of each coral attribute using the activation energy (*E*, in eV) as an effect size (Arrhenius, 1889; Gillooly et al., 2001; see supp. material for additional discussion of effect sizes). Impacts on coral attributes were extracted as observations in warming treatments (*V*_i_) relative to those in control (ambient) treatments (*V*_o_) under their corresponding temperature levels *T*_i_ and *T*_o_ respectively (where *T*_i_ > *T*_o_). These data permitted the derivation of *E* as an effect size per unit temperature under the assumption that the differences conform to an Arrhenius model and was calculated as,

(1) $$\text{Effect}\;\text{size}\left(E\right)=\frac{\ln V_{o}/V_{i}}{\frac{1}{kT_{o}}‐\frac{1}{kT_{i}}},$$

where *k* is the Boltzmann constant and *T* is the temperature (in Kelvin). We calculated *E* in this manner because rates derived as the slope of the Arrhenius equation could not be obtained from singular studies that typically employ one control treatment and one or two experimental treatments. Instead, *E* calculated in this manner, equivalent to a log ratio effect size (Hedges et al., 1999), allows for the derivation of a rate of change per unit temperature when all comparable experiments are pooled.

We quantified the sensitivity of each coral attribute to elevated CO_2_ using log ratio effect sizes (Ln RR), weighted by the extent of increase in *p*CO_2_ employed in individual experiments. We chose the Ln RR effect size over other methods (e.g. Hedges *g*′) to maintain symmetry in our analysis and for ease of interpretation of biological responses. Impacts on biological processes were extracted in a similar manner to the *E*. Mean responses to elevated CO_2_ (i.e. acidification, ${\bar{x}}_{\exp}$) were extracted relative to observations in control (ambient) treatments (${\bar{x}}_{\text{Cont}}$) under their respective *p*CO_2_ levels (in µatm). Although the data were extracted in a similar manner to *E*, the Ln RR effect size does not require the differences to conform to an Arrhenius model. These data permitted the derivation of an Ln RR as an effect size per unit *p*CO_2_ and was calculated as:

(2) $$\begin{array}{cc} \text{Effect}\;\text{size}\left(\text{Ln}\;\text{RR}\;\Delta \;100\;\mu {\text{atm}}^{‐1}\;{\text{CO}}_{2}\right) & =\frac{\text{Ln}\;\text{RR}}{\Delta p{\text{CO}}_{2}\times 100}\\ & =\frac{\left(\ln{\bar{x}}_{\exp}‐\ln{\bar{x}}_{\text{Cont}}\right)}{(p{\text{CO}}_{2\exp}‐p{\text{CO}}_{2\text{Cont}})\times 100}, \end{array}$$

where *p*CO_2Exp_ and *p*CO_2Cont_ are the *p*CO_2_ concentrations (in µatm) in experimental and control treatments respectively.

The calculation of effect sizes allows experimental assessments to be pooled across different species that may employ different approaches and methods, but measure a common effect. However, dimensionalities are removed when the results are calculated as effect sizes, therefore representing relative change, not absolute change, in performance. The power of effect size analyses to aggregate responses across species render these analyses a major corner stone of understanding the responses of marine organisms to heatwaves (Smale et al., 2019) and OA (Kroeker et al., 2010, 2013).

### 2.6 Determination of nature of combined effects

We quantified whether the responses of corals to the combined effects of warming and elevated *p*CO_2_ were additive or interactive (i.e. synergistic or antagonistic) in nature. The 248 independent full‐factorial experiments in the data set included four outcomes of experimental warming (${\bar{x}}_{\text{Warming}}$), elevated *p*CO_2_ (${\bar{x}}_{p{\text{CO}}_{2}}$), warming and elevated *p*CO_2_ (${\bar{x}}_{\text{Both}}$) and a control treatment (${\bar{x}}_{\text{Control}}$) to be included in the analysis. These data allowed for the determination of the interaction strength (Ln RR_Inter_) and individual effects (Ln RR_Warming_ and $\text{Ln}\;{\text{RR}}_{p{\text{CO}}_{2}}$) for each factorial experiment and were calculated according to methods for full‐factorial meta‐analyses (Gurevitch et al., 2000; Harvey et al., 2013):

(3) $$\text{Ln}\;{\text{RR}}_{\text{Inter}}=\frac{\left(\text{Ln}\;{\bar{x}}_{\text{Both}}‐\text{Ln}\;{\bar{x}}_{\text{Warming}}\right)‐\left(\text{Ln}\;{\bar{x}}_{p{\text{CO}}_{2}}‐\text{Ln}\;{\bar{x}}_{\text{Control}}\right)}{2s},$$

Then the individual effects of the stressors were calculated as:

(4) $$\text{Ln}\;{\text{RR}}_{\text{Warming}}=\frac{\left(\text{Ln}\;{\bar{x}}_{\text{Warming}}‐\text{Ln}\;{\bar{x}}_{\text{Cont}}\right)}{s},$$

(5) $$\text{Ln}\;{\text{RR}}_{p{\text{CO}}_{2}}=\frac{\left(\text{Ln}\;{\bar{x}}_{p{\text{CO}}_{2}}‐\text{Ln}\;{\bar{x}}_{\text{Cont}}\right)}{s},$$

where $\bar{x}$ is the mean biological response of the treatment (in subscript) and *s* is the pooled standard deviation calculated as:

(6) $$s=\frac{\sqrt{\left(\left(n_{\text{Both}}‐1\right)\times S_{\text{Both}}^{2}+\left(n_{\text{Warming}}‐1\right)\right.}\times \left.S_{\text{Warming}}^{2}+\left(n_{p{\text{CO}}_{2}}‐1\right)\times S_{p{\text{CO}}_{2}}^{2}+\left(n_{\text{Cont}}‐1\right)\times S_{\text{Cont}}^{2}\right)}{\left(n_{\text{Both}}+n_{\text{Warming}}+n_{p{\text{CO}}_{2}}+n_{\text{Cont}}‐4\right)}.$$

The sampling variance and 95% CI of Ln RR_Inter_ were calculated as:

(7) $$\begin{array}{cc} s_{N}^{2}\left(\text{Ln}\;{\text{RR}}_{\text{Inter}}\right) & =\frac{1}{n_{\text{Warming}}}+\frac{1}{n_{p{\text{CO}}_{2}}}+\frac{1}{n_{\text{Both}}}+\frac{1}{n_{\text{Cont}}}\\ & +\frac{\text{Ln}\;{\text{RR}}_{\text{Inter}}^{2}}{2\left(n_{\text{Warming}}+n_{p{\text{CO}}_{2}}+n_{\text{Both}}+n_{\text{Cont}}\right)}, \end{array}$$

(8) $${\text{CI}}_{\text{Ln}\;\text{RR}(\text{Inter})}\left(95\%\right)=\text{Ln}\;{\text{RR}}_{\text{Inter}}\pm 1.96\times s_{n}^{2}\left(\text{Ln}\;{\text{RR}}_{\text{Inter}}\right),$$

where *n* is the sample size of the treatment (in subscript).

This multiplicative model was used to calculate the nature of combined effects, because the underlying models of the effect sizes employed in this study (*E* and CO_2_ Ln RR) are multiplicative (Koricheva et al., 2013). The nature of combined effects of warming and elevated CO_2_ was considered to be additive if the 95% CI of the interaction effect size (Ln RR_Inter_) included zero. However, if the 95% CI of the Ln RR_Inter_ did not overlap with zero and the individual effect sizes of Ln RR_Warming_ and $\text{Ln}\;{\text{RR}}_{p{\text{CO}}_{2}}$ were either both negative or one negative and one positive, then the interaction was synergistic when the Ln RR_Inter_ was less than zero. Conversely, if the Ln RR_Inter_ was more than zero, the interactions were considered antagonistic (Harvey et al., 2013).

### 2.7 Meta‐analyses and sensitivity estimates

First, mixed‐effects meta‐analyses were performed on *E* estimates for each process to assess (i) sensitivity to warming at ambient CO_2_, and (ii) sensitivity to warming at elevated CO_2_. Second, two mixed‐effects meta‐analyses were conducted on Ln RR (Δ100 µatm^−1^) estimates for each coral attribute to assess (i) the effect of elevated CO_2_ at ambient temperature, and (ii) the effect of elevated CO_2_ at elevated temperature (Table S4). All mixed‐effects meta‐analyses were conducted using the rma.mv function from the metafor package in R (Viechtbauer, 2010). Meta‐analyses were weighted for variance of the individual experiments and included two random variables. We included study identification and coral genus as random variables to minimize potential bias from dependence among measures and eliminate bias towards widely distributed species commonly assessed in our data set (e.g. *Acropora* spp.). Mean effect size estimates were significant if they differed from zero (*p* < 0.05; Table S4).

Based on the results of the meta‐analyses, we estimate the response of the coral attributes under predicted temperature and CO_2_ increases as per cent changes relative to responses under present‐day conditions. A per cent change of zero indicates that the coral attribute will operate at rates similar to those observed under present‐day conditions, whereas a −50% decline indicates that rates will operate at approximately half the rate relative to present‐day responses. We calculated the minimum increase in treatment level (in temperature °C and CO_2_ µatm) required to cause a significant per cent change in the coral attribute responses. For this, *E* and Ln RR CO_2_ effect sizes from the meta‐analyses (presented in Figure 2a,c; Table S4) were back‐transformed to mean per cent change estimates using Equations (9) and (10), respectively, and were considered significant when a given increase in treatment level resulted in the back‐transformed 95% CI bounds not overlapping with zero (Table S4). For this, we applied a loop in *R* to repeatedly calculate per cent change estimates at incremental treatment levels until they surpassed the chosen level (i.e. when the CI did not include zero). The temperature and CO_2_ increases required to produce the least significant per cent change for each coral attribute were reported as estimates of statistical sensitivity to changes in temperature and CO_2_.

(9) $$\text{Expected}\;\text{change}\left(\%\right)=\left(100‐e^{\left(\text{Estimate}[E]\times \Delta 1/kT\right)}\right)\times 100,$$

where 1/*kT* values corresponding to temperature increases in °C were obtained from the calculated linear relationship between temperature increases in Celsius and 1/*kT* values in the data set.

(10) $$\text{Expected}\;\text{change}\left(\%\right)=100‐\left(e^{\left(\text{Estimate}\left[{\text{CO}}_{2}\text{Ln}\;\text{RR}\right]\times \Delta {\text{CO}}_{2}/100\right)}\right)\times 100,$$

where *∆*CO_2_ is the increase in the partial pressure of CO_2_ (in µatm).

For each coral attribute, we calculated per cent changes at future MHW intensities projected under three main RCPs (2.6, 4.5 and 8.5) for the near‐term (2021–2040), mid‐century (2041–2060) and late‐century (2081–2100) relative to present‐day responses (control) using Equation (9). Temperature increases for each MHW scenario represent the MHW intensities exceeding the 90th pre‐industrial percentile (Table S1). Likewise, we calculated expected response ratios for end‐of‐century (2091–2100) CO_2_ increases projected under RCP2.6 (+63 µatm), RCP4.5 (+173 µatm), RCP6.0 (+276 µatm) and RCP8.5 (+490 µatm), relative to present‐day responses (control) according to Equation (10). For all coral attributes, estimates of per cent change were considered significant when 95% CI bounds did not include zero.

### 2.8 Publication bias

Publication bias refers to the influence of selective publications to potentially skew or distort the results of a meta‐analysis (Koricheva et al., 2013). In this study, selective publications could lead to an under‐ or overestimation of the effects of MHW and OA scenarios (i.e. warming and OA treatments) on coral attribute performance. We assessed the potential for publication bias by interpreting funnel plots and forest plots using the funnel (Sterne & Egger, 2001) and forest (Lewis & Clarke, 2001) functions from the metafor package in R (Viechtbauer, 2010). If any outliers were observed, studies were removed individually and the mixed‐effects meta‐analyses were rerun to assess whether the outcome of the analysis (i.e. significance to *p* < 0.05) changed. Visual observation of plots (observations outside 95% CI of funnel plots or study CI not overlapping with overall model CI in forest plots) revealed that 13 out of the 32 meta‐analyses may be biased by individual publications. Adjusting for outlying results did not affect the outcome of these analyses, except for three that assessed the sensitivity of photosynthesis and calcification in response to warming alone, and the sensitivity of photosynthesis to warming at elevated *p*CO_2_. However, we retained the identified studies (Bahr et al., 2016; Camp et al., 2016; Noonan & Fabricius, 2015) in the analyses because details of these studies indicate robust experimental approaches that closely mimic natural settings. We retained these studies in the analysis since (i) there was no evidence to indicate that responses reported fell outside natural variation, and (ii) because removing these studies did not affect our overall conclusions.

## 3 RESULTS

### 3.1 Projections of MHWs under global warming scenarios

**FIGURE 1:**
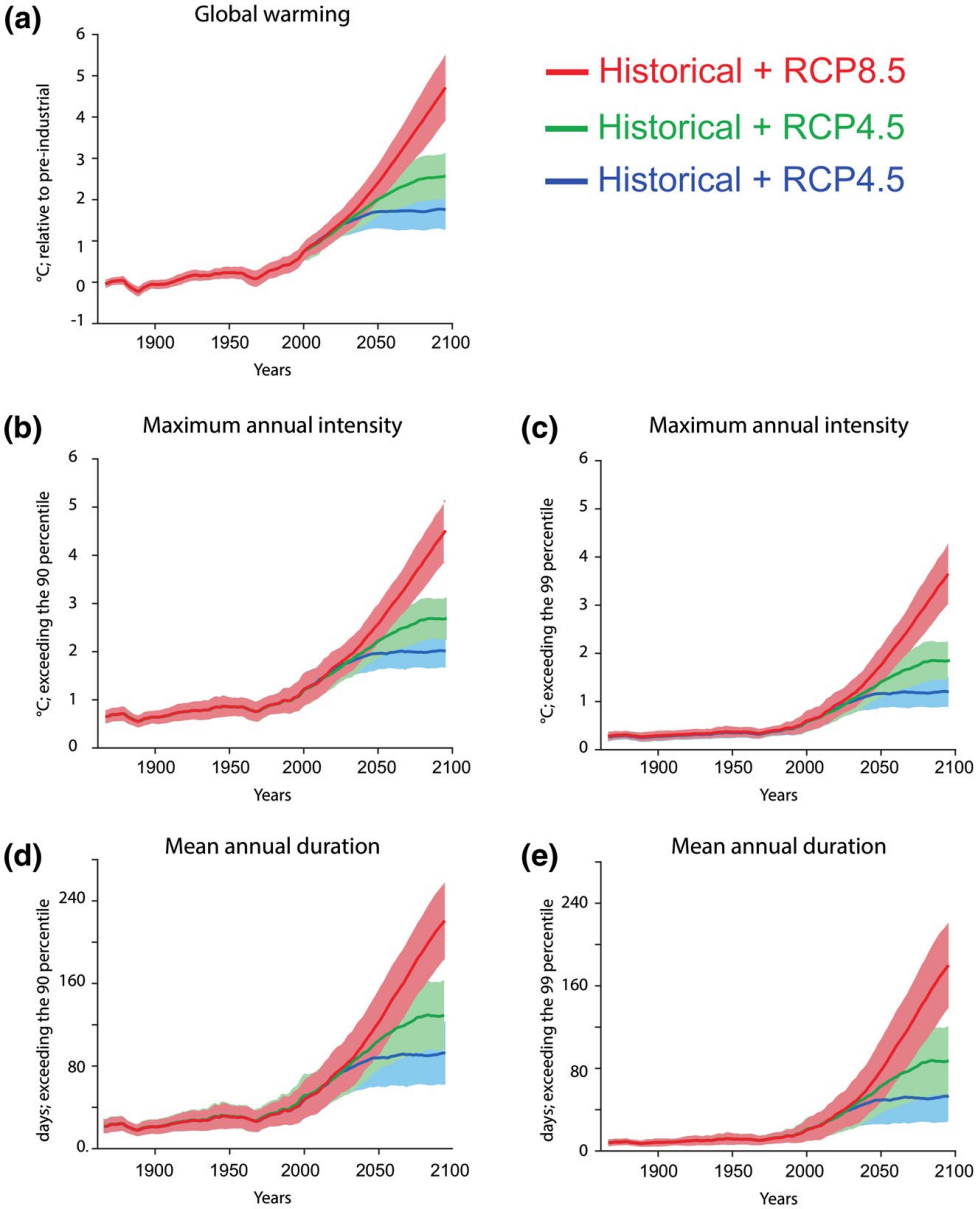
Simulated changes in the intensity and duration of marine heatwaves (MHWs) under different global warming scenarios. Time‐series simulations are shown for (a) simulated global mean atmospheric surface temperature relative to pre‐industrial (1861–1880), (b) maximum annual intensity of MHWs exceeding the 90th pre‐industrial percentile, (c) maximum annual intensity of MHWs exceeding the 99th pre‐industrial percentile, (d) mean annual duration of MHWs exceeding the 90th pre‐industrial percentile and (e) mean annual duration of MHWs exceeding the 99th pre‐industrial percentile. In all panels, the thick lines represent the multi‐model averages and the shaded plumes represent the minimum and maximum values for the RCP2.6, RCP4.5 and RCP8.5 scenarios (cf. Section 2) for tropical and subtropical between +30 and −30 degrees latitude. RCP, representative concentration pathway

We first calculated maximum annual MHW intensities and the annual mean duration of MHWs from simulations of 12 Earth system models for tropical and subtropical regions (between +30 and −30 degrees of latitude) under three (low, intermediate and high) RCPs (2.6, 4.5 and 8.5) for the near‐term (2021–2040), mid‐century (2041–2060) and late‐century (2081–2100; Figure 1; Table S1). The intensity of MHWs (defined relative to the 90th percentile of pre‐industrial control simulations) increased from 0.68°C in 1861–1880 to 1.2°C in 1991–2010 (Figure 1b), and the number of MHW days increased from 23 to 41 over the same time interval (Figure 1d). This trend is projected to accelerate in the future, regardless of whether MHWs were defined relative to the 90th or 99th percentile (Figure 1; Figure S1; Table S1). By the end of the 21st century, the maximal intensity of MHWs is projected to increase by a factor of 4 under an intermediate emissions scenario (RCP4.5) and by a factor of 6 under a high emissions scenario (RCP8.5), relative to the 90th percentile pre‐industrial control simulations.

### 3.2 Coral sensitivity to heatwaves

**FIGURE 2:**
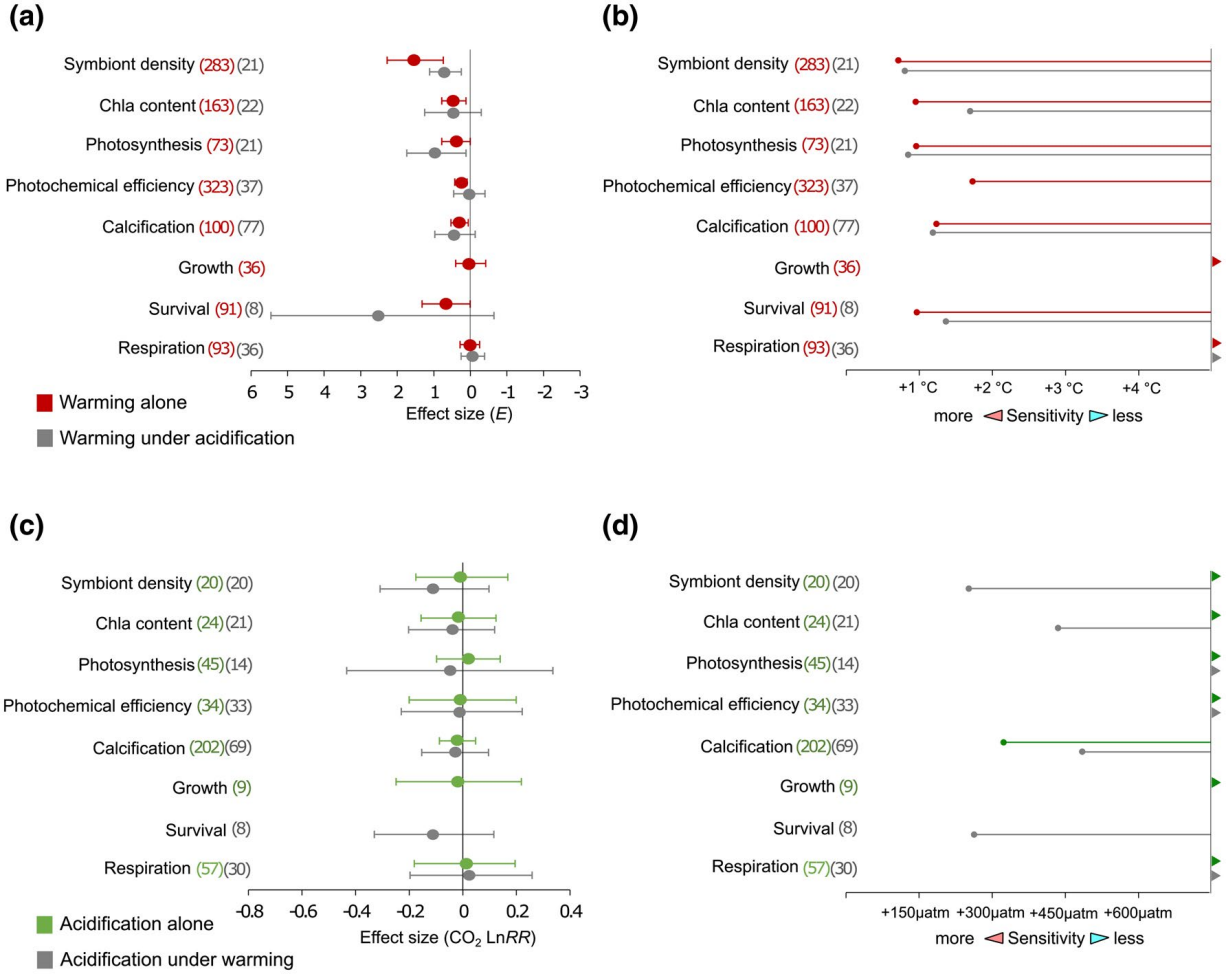
Extent of coral attribute sensitivity depends on effect size parameterization (*E* vs. CO_2_ Ln RR) and the presence of a parallel stressor. (a) Mean activation energy effect size estimates (*E* ± 95% CI) of attribute sensitivity to warming depends on the coral attribute type and the presence of acidification. (b) Statistical sensitivity estimates represent the minimum temperature change (degrees Celsius, °C) required to produce a significant per cent change in the coral attribute response when exposed to warming alone (red lines) and warming under acidification (dark grey lines). (c) Mean CO_2_ log response ratio effect size estimates (Ln RR CO_2_ ± 95% CI) of attribute sensitivity to acidification alone and acidification in the presence of warming. (d) Sensitivity estimates represent the minimum change in seawater acidification (µatm CO_2_) required to produce a significant per cent change in the coral attribute response when exposed to acidification alone (green lines) and acidification in the presence of warming (dark grey lines). (a, c) Effect size estimates to the right of the solid lines (at zero) indicate increases in rate responses and those to the left denote decreases in responses. For each effect size, the number of observations are specified in parentheses (b) and (d), lines for each attribute end where significant effects were observed and attributes with greater sensitivity have longer lines. Lines that end with arrows indicate that the minimum temperature change required to cause a significant per cent change was less than 0.5°C. Estimates of statistical sensitivity were derived from mean estimate effect size estimates and 95% CI boundaries presented in panels (a) and (c) (cf. Section 2)

**FIGURE 3:**
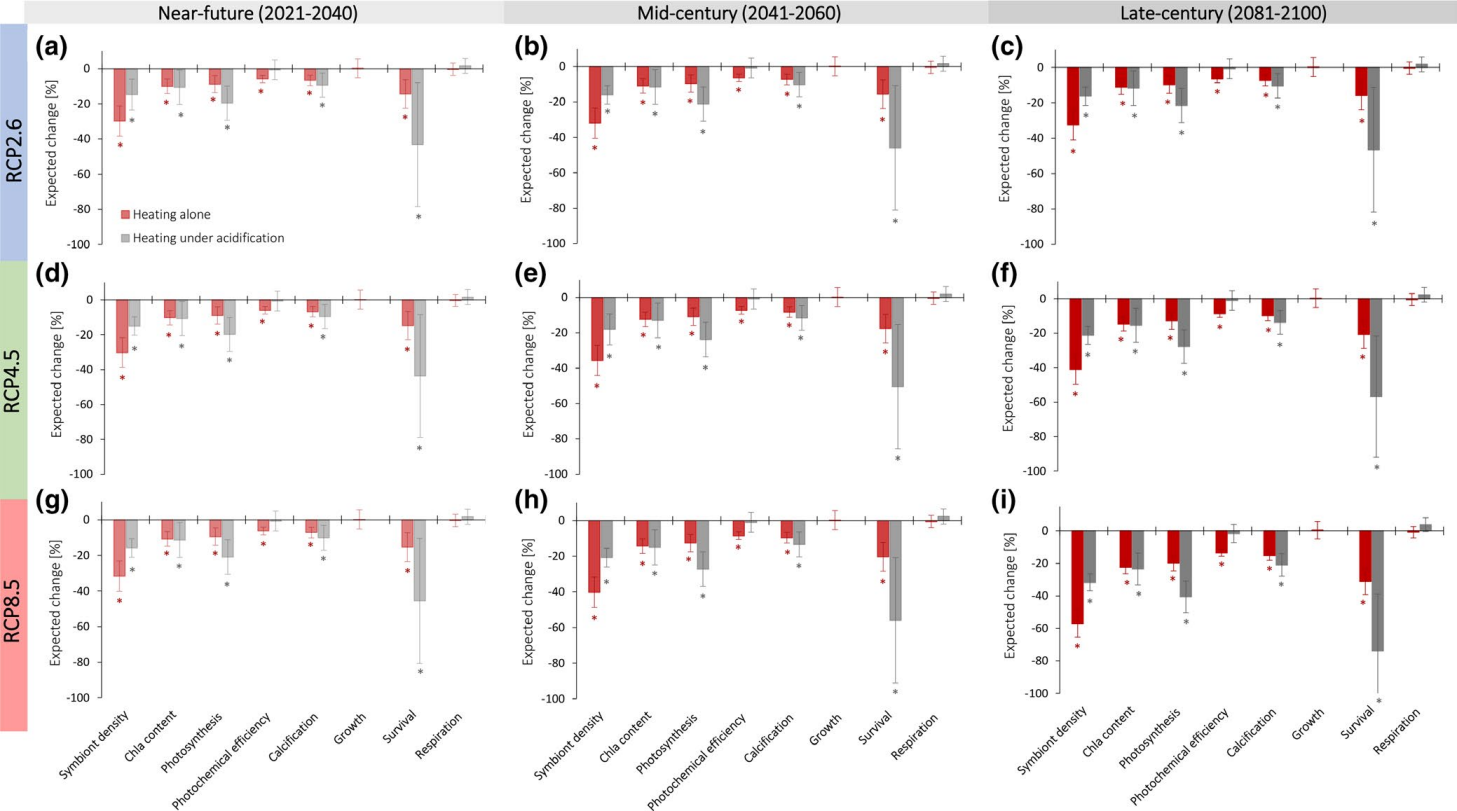
Declines in the future coral performance and survival under increasing marine heatwave (MHW) intensities throughout this century. (a–i) Mean estimates of expected decreases (percentage ± 95% CI) relative to experimental controls. Horizontal panels display expected attribute decreases for RCPs 2.6 (a–c), 4.5 (d–f) and 8.5 (g–i), and vertical panels show performance for near‐term (2021–2040, a, d and g), mid‐century (2041–2060, b, e and h) and late‐century (2081–2100, c, f and i). Red bars represent percentage decreases in response to future temperature increases and grey bars represent decreases in response to future temperature increases, but in the presence of ocean acidification. Mean expected decreases were derived from *E* effect size estimates presented in Figure 2 and parameterized by future temperature increases for each scenario (a–i). Future temperature increases for each scenario (a–i) represent the combined temperature increases projected for persistent global warming and future MHW intensities exceeding the 90th pre‐industrial percentile (Table S1). Bars denoted with * represent significant decreases, determined by when the 95% CI did not overlap with zero (no decrease). RCP, representative concentration pathway

Our analysis (based on experimental effect sizes parameterized by expected levels of warming) confirms that coral performance and survival will deteriorate under future MHWs (Figures 2 and 3). However, the projected effect of MHWs is dependent on the focal coral performance attribute as temperatures increase throughout this century (Figures 2 and 3; Table S4). Symbiont density and survival are perhaps the most vital attributes among those examined in our analysis because they directly reflect a lethal limit and the systematic breakdown of the coral holobiont association. These attributes were coincidentally the most thermally sensitive (*E* [mean ± 95% CI] = 1.5 ± 0.8 and 0.7 ± 0.7 eV respectively). We assessed the statistical sensitivity of the effect size from the meta‐analyses (Figure 2a,c) by calculating the minimum increase in temperature required to cause a significant per cent change in the coral response attribute. Both symbiont density and survival showed significant declines of 8% under less than 1°C warming (Figure 2b). The ability of corals to build their hard skeleton (coral calcification) was less sensitive and showed an initial decline of 3% at 1.3°C, closely matching the initial sensitivity of estimates for symbiont‐measured attributes (chl*a* content, photosynthesis and photochemical efficiency), which all declined by less than 5% under <1°C of warming (Figure 2b). In contrast, oxygen demand of the holobiont (respiration) and coral growth were seemingly unaffected by warming exceeding 5°C (Figure 2b).

### 3.3 Coral sensitivity to OA

**FIGURE 4:**
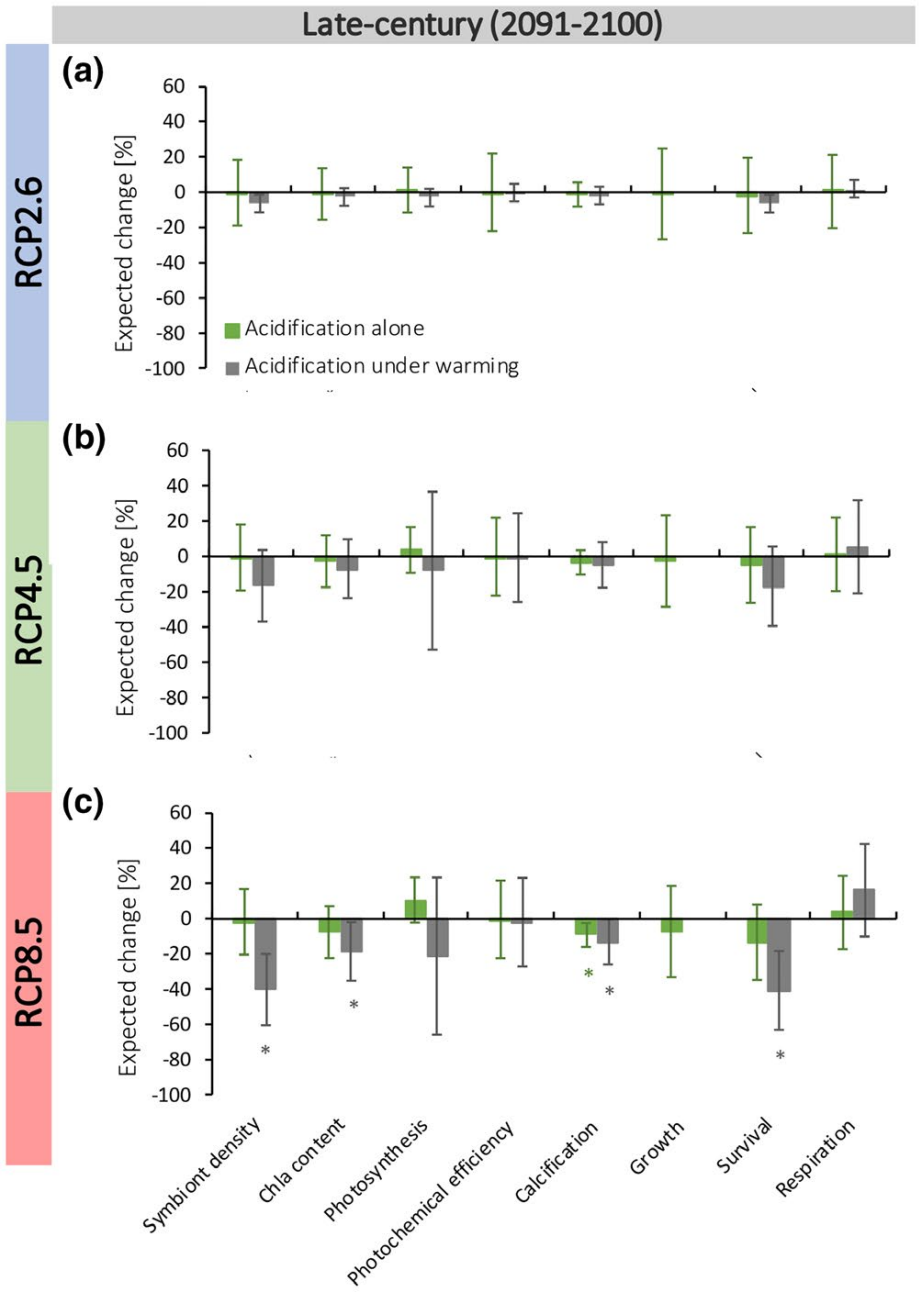
Estimates of future coral performance parameterized by end‐of‐century ocean (2091–2100) acidification. (a–c) Mean estimates of expected decreases (percentage ± 95% CI) relative to experimental controls for RCPs 2.6 (a), 4.5 (b) and 8.5 (c). Negative per cent change estimates represent an expected decrease in the attribute, whereas a positive per cent change indicates an expected increase. Green bars represent percentage change of coral attributes under future ocean acidification and grey bars also represent in response to future ocean acidification, but in the presence of warming. (a–c) Mean expected decreases were derived from CO_2_ Ln RR effect size estimates presented in Figure 2c and parameterized by future seawater CO_2_ increases for each RCP scenario. Future CO_2_ increases for each scenario (+63 µatm [RCP2.6], +173 µatm [RCP4.5] and +490 µatm [RCP8.5]) represent expected increases above present‐day levels by the end of this century (2091–2100, cf. Section 2). RCP, representative concentration pathway

To understand the influence of OA on coral performance and survival, we first assessed acidification as a singular driver using log response ratio (Ln RR) effect sizes parameterized by increases in the partial pressure of CO_2_ (Figures 2c and 4). Coral performance and survival were largely unaffected by any OA scenario tested in our analysis (Figures 2d and 4), with the exception of coral calcification, which declined by 9.2% under the most pessimistic end‐of‐century OA scenario (RCP8.5, +490 µatm CO_2_; Figure 4c). Overall, our meta‐analysis of coral attribute responses to OA produced estimates with substantial variability (Figures 2c and 4), reflecting a diversity of effects of OA on coral performance and survival. In contrast, the variability associated with most of our thermal sensitivity estimates was much smaller (Figures 2a and 3), indicating that future coral performance will be more dependent on intensifying temperatures than on OA.

Among the seven coral attributes for which sufficient data were available in response to the dual stressors (Figure 2a), the amount of warming required to produce initial, significant declines in symbiont density, calcification and photosynthesis remained similar whether OA was concomitant (Figure 2b). By contrast, the warming required to induce changes in chl*a* content, photochemical efficiency and coral survival differed under co‐occurring OA (Figure 2b). However, there was a clear discrepancy between the role of OA in affecting the initial, statistical sensitivity of attribute estimates to warming (Figure 2b) and their overall performance under the future MHW scenarios (Figure 3). For instance, photosynthesis initially declined at 0.97°C of warming alone and declined at 0.84°C of warming when occurring with acidification. Despite this similarity, the addition of OA led to reductions in mean estimates for photosynthesis under the late‐century RCP4.5 scenario (Figure 3f), as well as the mid and late‐century RCP8.5 scenarios (Figure 3h,i). On the other hand, mean estimates for calcification first declined at 0.77°C of warming alone, but this sensitivity estimate extended to 1.31°C of warming along with OA. However, projected declines in calcification were similar regardless of the presence OA under all MHW scenarios (Figure 3). In the case of coral survival, 0.97°C of warming alone caused a significant decline, but survival did not decline until 1.38°C with both drivers (Figure 2b). Despite this, the addition of OA led to substantial reductions in coral survival (74%) that were more than twofold greater than those expected if MHWs were the sole driver (31%) under the most pessimistic heatwave scenario (+4.32°C under RCP8.5) for the late century. Although this pattern was consistent across all MHW scenarios (Figure 3), there was no statistical difference between survival estimates under warming alone and warming with concomitant acidification for all other scenarios (Figure 3). Overall, intensifying temperatures and OA had predominately additive effects (78%) on coral responses and only a small fraction of responses reflected antagonistic (16%) or synergistic (6%) interactions of the dual stressors.

### 3.4 Projections of coral performance under MHWs in an acidifying future ocean

Given that ocean warming and OA will progress in parallel, we primarily focus on future responses parameterized by projected MHW conditions in the presence of OA. In the near‐term (2021–2040), we calculated that symbiont density will decline by 15% when MHW temperatures reach +1.8℃ under the low emissions scenario (RCP2.6) and decline by 16% at +1.94°C under RCP8.5 (Figure 3a,d,g). The near‐term scenarios led to declines in chl*a* content that were slightly less than those expected for symbiont density (10%–11%; Figure 3a,d,g). However, declines in photosynthesis ranged from 20% up to 21% for the three scenarios, reflecting the proportional impact of symbiont and pigment loss on coral productivity (Figure 3a,d,g). In addition, coral calcification declined by between 9% and 10%, implicating intensifying MHWs as a key driver of reduced reef‐building capacity in the near‐term. Mean per cent declines in coral survival were the most profound across the near‐term scenarios and ranged between 43% and 46% (RCP2.6–RCP8.5; Figure 3a,d,g). Despite the variability in survival estimates, statistical significance under MHW scenarios indicate acute and potentially irreversible coral losses within the next two decades. This conclusion holds even if immediate efforts are made to limit global warming to 2°C above pre‐industrial levels (RCP2.6: Figure 3a).

By mid‐century (2041–2060), trajectories of MHW intensities clearly diverge among the three scenarios and the consequences of failing to curb anthropogenic emissions on coral responses become more apparent over time (Figure 1b,c), with the largest declines under RCP8.5 towards the end of this century (Figure 3h,i). Mean per cent reductions in symbiont density worsen to 16% under RCP2.6 by mid‐century (+1.96°C) and extend up to 21% under RCP8.5 (+2.63°C), with slightly larger reductions expected in photosynthesis, ranging between 21% and 27% among the mid‐century scenarios (Figure 3b,e,h). The mid‐century scenarios also resulted in further declines in chl*a* content and calcification, but mean losses in coral survival were most severe, reaching up to 56% under RCP8.5 (Figure 3b,e,h). Towards the end of the century, MHW scenarios simulated for RCP8.5 (+4.32°C) caused the steepest declines in coral performance, whereas RCP2.6 scenarios resulted in more steady declines across time. Despite this, our analysis indicates that MHWs under RCP2.6 will still lead to substantial impacts on coral productivity, reef‐building capacity and most importantly their survival (Figure 3c). In a scenario where unrestricted emissions continue to intensify (RCP8.5), these impacts become extreme with declines in coral survival exceeding 70% towards the end of this century (Figure 3i).

## 4 DISCUSSION

Our analysis shows that most symbiont‐related attributes and, consequently, coral survival are sensitive to the future MHW scenarios (in the presence and absence of OA) tested in our analysis. However, the notion that the sensitivity of symbiont communities alone may ultimately determine the ability of a coral to withstand heat stress has been recently challenged (Barshis et al., 2013). Corals themselves likely play a fundamental role in shaping symbiont‐measured attributes (Barshis et al., 2013; Hawkins et al., 2016), suggesting that coral responses to heat stress are a product of interactions between the partners involved, reflected across our attributes. The apparent resilience of respiration to warming alone and under higher CO_2_ levels may have resulted from a net balance between mixed responses among corals, and the relative contributions of individual partners within the holobiont that could offset one another (Hawkins et al., 2016), especially when algal symbiont contributions dissipate during bleaching. While most measures of thermal performance curves typically show a unimodal shape, performance curves of coral respiration appear to be particularly asymmetric, with a steady increase along an increasing thermal gradient (Anton et al., 2020; Jurriaans & Hoogenboom, 2019), followed by a quick decline preceding the critical lethal threshold (Jurriaans & Hoogenboom, 2019). Hence, the directional effect of warming on holobiont oxygen demand fundamentally depends on where individual corals are positioned along this performance curve. Indeed, moderate changes in some coral attributes may not necessarily translate into negative fitness consequences, but could also signify acclimatization processes (e.g. plasticity in coral respiration rates; Herrera et al., 2020). In the case of host growth, warm‐water corals can sustain uninhibited growth and extension rates despite inhibited calcification rates by enhancing skeletal porosity; however, this increases coral fragility (Fantazzini et al., 2015). Although our analysis indicates that holobiont respiration and coral growth were resilient to the future scenarios, it is likely that the reef‐building capacity of corals and their metabolism will be affected by future MHWs and OA.

Across all MHW scenarios (Figure 3), our findings show declines in symbiont density, productivity, calcification and coral survival, which is consistent with past and present‐day observations of heatwaves on coral reefs, where heat stress induces widespread coral bleaching often resulting in substantial coral loss (Baker et al., 2008; Spalding & Brown, 2015). Even though our projections characterize the impact of future ocean conditions based on separate holobiont attributes, the sensitivity of the coral–algal symbiosis itself exceeds that of either partner. For instance, reduced algal symbiont densities will directly translate into reduced photosynthesis, resulting in reduced resource supply to the host. Hence, attributes reflect the state of the holobiont rather than the state of individual partners. A more in‐depth understanding of coral susceptibility to MHWs and OA will therefore require disentangling symbiotic interactions within the holobiont complex. Even so, the most frequently measured attributes in our analysis reflect performance measures of either host (e.g. calcification) or symbiont (e.g. photosynthesis, photochemical yield), rather than direct assessments of the interaction between the two. Future assessments quantifying the parallel impacts of MHWs and OA on symbiotic interactions (e.g. carbon translocation, oxidative stress, signalling or immune responses) are urgently needed to advance our understanding of the impacts of climate change on corals as complex associations.

Globally, MHWs are becoming more intense, frequent and prolonged as well as affecting wider geographic areas (Frölicher et al., 2018; Oliver et al., 2018). Our analysis captures the consequences of intensifying MHW temperatures and delivers a bleak outlook for corals under future warming and OA. However, we could not assess the effect of increasing MHW duration on coral performance and survival (Figure 1d,e). The heatwaves simulated by the experiments used in our analysis lasted 22 days on average, substantially shorter than the simulated duration for MHWs throughout this century, although Earth system models also have difficulties in correctly representing MHW duration (Pilo et al., 2019). According to our 90th percentile simulations, MHWs will have a mean duration of 49 days by mid‐century under the low emissions scenario RCP2.6 and extend to 82 days under the high emissions scenario RCP8.5 for the same time period (Figure 1d; Table S1). This is especially concerning in the context of the 2015/16 global bleaching event, where millions of coral colonies died on the Great Barrier Reef after only 14–21 days of exposure to a MHW (Hughes, Kerry, et al., 2018). Finally, we did not assess expected impacts on coral reproductive efforts and spawning rates because these responses are rarely measured in experimental manipulations. Hence, the attributes we used to project coral futures do not provide a comprehensive assessment across all life‐history traits, but focus on the physiological performance and survival of mature corals.

Our results provide a generalized impact of MHWs and OA on coral performance and survival according to a single, pantropical parameterization, but regional differences may exist. For instance, corals located in the western Pacific and Coral Triangle show the greatest resistance to thermal stress, whereas corals outside these ecoregions exhibit greater vulnerability to thermal stress (McClanahan et al., 2020). Downscaling our pantropical projections to resolve regional differences is, however, precluded by a lack of experiments testing regional coral responses to warming and increased *p*CO_2_. We, therefore, encourage efforts to resolve these responses at regional scales.

Crucially, our estimates of future coral performance and survival could be affected by publishing bias when experiments with minimal or no effects are less likely to be published. Indeed, the potential issue of publication bias applies to almost all meta‐analyses, but is a crucial limitation in sensitivity assessments. This issue maybe particularly likely for experiments with minimal warming and acidification levels, where undetectable effects might be common. Although Harvey et al., (2013) reported no evidence of such publication bias in an assessment of marine taxa responses to warming and OA (Harvey et al., 2013), we emphasize the need for studies that report non‐significant effects to be published in peer‐reviewed journals. Uncertainties may also arise from pooling experimental assessments that can differ in their underlying approaches (e.g. experimental duration, feeding and light regimes).

Coral reefs as we know them today will undoubtedly undergo substantial changes in ecological structure and functioning. Climate science has taken great strides in simulating accurate trends for weather extremes under various scenarios of anthropogenic climate change (Cai et al., 2015; Coumou & Rahmstorf, 2012), providing essential insight into the dramatic impacts likely to come. Our projections of coral performance and survival show that although warming and acidification had predominately additive impacts on coral responses, the contribution of OA in affecting most coral attributes appears small relative to the dominant role of intensifying MHWs. In addition, our findings indicate that impact of OA and intensifying MHW temperatures is specific to the coral attribute examined and the extremity of temperatures tested. These projections of coral performance paint a grim picture in which the future scenarios will cause worsening instances of coral bleaching, reduced productivity and mortality over the coming decades. However, our analysis does not account for genetic adaptation, acclimatization or adaptive selection potential, which could reduce coral vulnerability. Indeed, a recent study of coral bleaching events across the tropics showed that despite increases in the frequency and intensity of MHWs over the past decade, the onset of coral bleaching occurred at higher SSTs (~0.5°C) than in the previous decade (Sully et al., 2019). This increase in bleaching threshold likely occurred via the selection of thermally resilient genotypes from standing genetic diversity and/or the acclimatization of more susceptible genotypes (Sully et al., 2019). However, it remains uncertain whether acclimatization, genetic adaptation and/or selection processes can further increase coral resilience as MHWs become longer and occur more often.

## CONFLICT OF INTEREST

The authors declare no competing interests.

## AUTHOR CONTRIBUTIONS

Shannon G. Klein, Nathan R. Geraldi, Andrea Anton, Sebastian Schmidt‐Roach, Maren Ziegler and Carlos. M. Duarte conceptualized this study. Shannon G. Klein, Nathan R. Geraldi, Andrea Anton, Sebastian Schmidt‐Roach, Maren Ziegler, Maha J. Cziesielski, Nils Rädecker, Cecilia Martin, David J. Suggett, John M. Pandolfi, Peter J. Mumby, Christian R. Voolstra, Manuel Aranda and Carlos. M. Duarte participated in a workshop that designed this study. Thomas L. Frölicher performed the MHW analysis. Shannon G. Klein, Nathan R. Geraldi, Andrea Anton, Sebastian Schmidt‐Roach, Maren Ziegler, Maha J. Cziesielski, Cecilia Martin and Nils Rädecker extracted the coral metadata. Nathan R. Geraldi, Shannon G. Klein and Andrea Anton reduced the coral metadata set and conducted the meta‐analyses. Shannon G. Klein, Sebastian Schmidt‐Roach, Thomas L. Frölicher and Carlos. M. Duarte designed and produced figures. Shannon G. Klein, Nathan R. Geraldi, Andrea Anton, Sebastian Schmidt‐Roach, Maren Ziegler, Nils Rädecker, Manuel Aranda and Carlos. M. Duarte interpreted the data. Shannon G. Klein and Carlos. M. Duarte wrote the initial manuscript with contributions from Sebastian Schmidt‐Roach and Nils Rädecker, and all authors contributed to, and approved, the final version.

## Supporting information

Supplementary Material

## Data Availability

All source meta‐data files of coral attribute responses are available from https://github.com/ngeraldi/cnidarian_meta‐analysis. The code supporting the meta‐analyses is available at https://github.com/ngeraldi/cnidarian_meta‐analysis.
