# Supplementary material for: Projecting coral responses to intensifying marine heatwaves under ocean acidification
Source: Glob Chang Biol. 2021 Aug 29;28(5):1753–65. doi: 10.1111/gcb.15818 (PMC9291544; doi:10.1111/gcb.15818)
Supplement: Supplementary file 1 — Supplementary Material [file GCB-28-1753-s001.docx]

**Supplementary Information for**

Projecting coral responses to intensifying marine heatwaves under ocean acidification

Shannon G. Klein^1^*, Nathan R. Geraldi^1^, Andrea Anton^1^, Sebastian Schmidt-Roach^2^, Maren Ziegler^2,3^, Maha J. Cziesielski^2^, Cecilia Martin^1^, Nils Rädecker^2^, Thomas L. Frölicher^4,5^, Peter J. Mumby^6^, John M. Pandolfi^7^, David J. Suggett^8^, Christian R. Voolstra^2,9^, Manuel Aranda^2^, and Carlos. M. Duarte^1^.

^1^ King Abdullah University of Science and Technology (KAUST), Red Sea Research Center (RSRC) and Computational Bioscience Research Center (CBRC), Thuwal, 23955-6900, Kingdom of Saudi Arabia

^2^ King Abdullah University of Science and Technology (KAUST), Red Sea Research Center (RSRC), Thuwal, 23955-6900, Kingdom of Saudi Arabia

^3^ Department of Animal Ecology & Systematics, Justus Liebig University, Heinrich-Buff-Ring 26-32 IFZ, D-35392 Giessen, Germany

^4^ Climate and Environmental Physics, Physics Institute, University of Bern, Bern, Switzerland

^5^ Oeschger Centre for Climate Change Research, University of Bern, Bern, Switzerland

^6^ Marine Spatial Ecology Lab, School of Biological Sciences, The University of Queensland, St. Lucia, Brisbane, QLD 4072, Australia

^7^Australian Research Council Centre of Excellence for Coral Reef Studies, School of Biological Sciences, The University of Queensland, St. Lucia, Brisbane, QLD 4072, Australia

^8^ University of Technology Sydney, Climate Change Cluster, Faculty of Science, Sydney, NSW 2007, Australia

^9^ Department of Biology, University of Konstanz, Konstanz 78457, Germany

*** Correspondence**: *Shannon G. Klein **Email:**  [shannon.kein@kaust.edu.sa](mailto:shannon.kein@kaust.edu.sa)

**This PDF file includes:**

Additional discussion of effect sizes

Figures S1 to S5

Tables S1 to S5

**Additional discussion of effect sizes used to parametrize coral attribute responses**

The effect size used to parametrize coral attribute responses by rates of warming (i.e. marine heatwave intensities) was calculated using the activation energy, which is based on Arrhenius law(Arrhenius, 1889). The activation energy (*E*, expressed as eV [Electron Volts]) captures the predictable and governing influence of temperature on terrestrial, freshwater (Gillooly, Brown, West, Savage, & Charnov, 2001), and marine animals (Kordas, Harley, & O'Connor, 2011) by estimating the strength and direction (e.g., increases or decreases) of changes in biological processes with warming - termed ‘thermal sensitivity’ here. Mathematically, *E* is equivalent to a log effect size estimate (Hedges, Gurevitch, & Curtis, 1999) that can be used to measure the strength and direction with the extent of heating tested. The Arrhenius law forms the backbone of metabolic theory of ecology (Brown, Gillooly, Allen, Savage, & West, 2004), which has demonstrated the predictive power of *E* across a range of organisms and environments (Canavero, Arim, Pérez, Jaksic, & Marquet, 2018; Gibert, Chelini, Rosenthal, & DeLong, 2016; Savva, Bennett, Roca, Jordà, & Marbà, 2018). Although few coral studies have adopted *E* as a projecting tool, it has been shown to reliably project the thermal tolerance of a diverse range of coral symbiont taxa (Anton et al., 2020; Wang, Meng, Chen, & Chen, 2012; Wang, Wang, Keshavmurthy, Meng, & Chen, 2019) and described the thermal sensitivity of growth rates (Clausen, 1971; Weber & White, 1974) and enzyme activity (Wang et al., 2019) across numerous coral species. Although no equivalent body of theory exists to estimate the changes in physiological performance to different extents of seawater acidification, we used the log effect size (LnRR 100μatm^-1^ *p*CO_2_) to measure the experimental effect of acidification on coral attributes (Chan & Connolly, 2013; Wittmann & Pörtner, 2013).


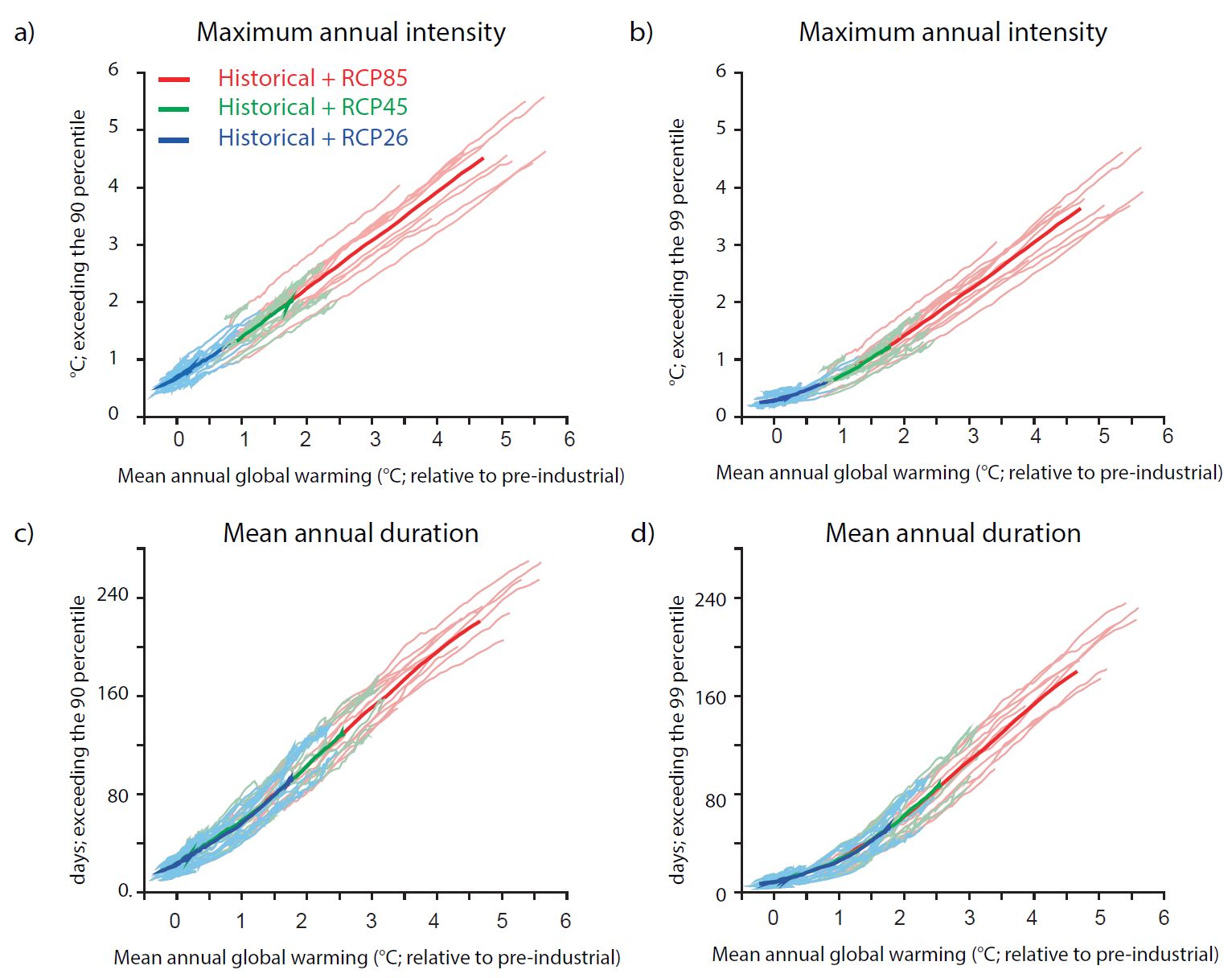


Figure S1. Simulated relationship between global warming and the intensity and duration of MHWs under different global warming scenarios. Simulated relationship between simulated global mean atmospheric surface temperature relative to pre-industrial (1861–1880) and (a) maximum annual intensity of MHWs exceeding the 90th preindustrial percentile, (b) maximum annual intensity of MHWs exceeding the 99th preindustrial percentile, (c) mean annual duration of MHWs exceeding the 90th preindustrial percentile, and (d) mean annual duration of MHWs exceeding the 99th preindustrial percentile. In all panels, the thick lines represent the multi-model averages and the thinner lines represent individual model projections for the RCP2.6, RCP4.5, and RCP 8.5 scenarios (cf. Supplementary methods) for ±30 degrees latitude.


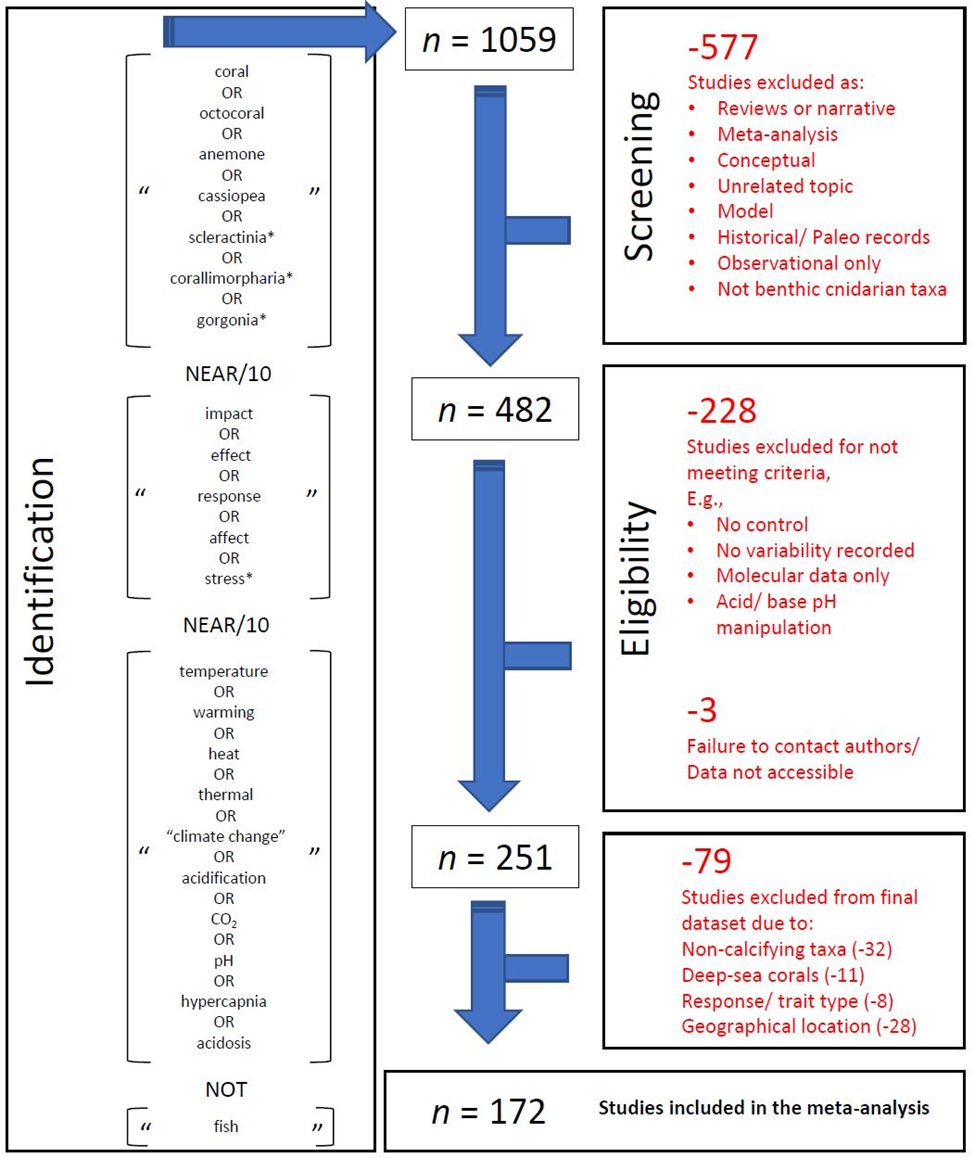


**Figure S2. Flowchart for the publication selection process based on PRISMA (Preferred Reporting Items for Meta-analyses) guidelines.** Flowchart depicts the selection process followed to include publications in the meta-analysis. Chart format is based on example provided by PRISMA website (http://www.prisma-statement.org/). Search term listed in ‘Identification’ panel was used in the Web of Science (WoS) database.

**
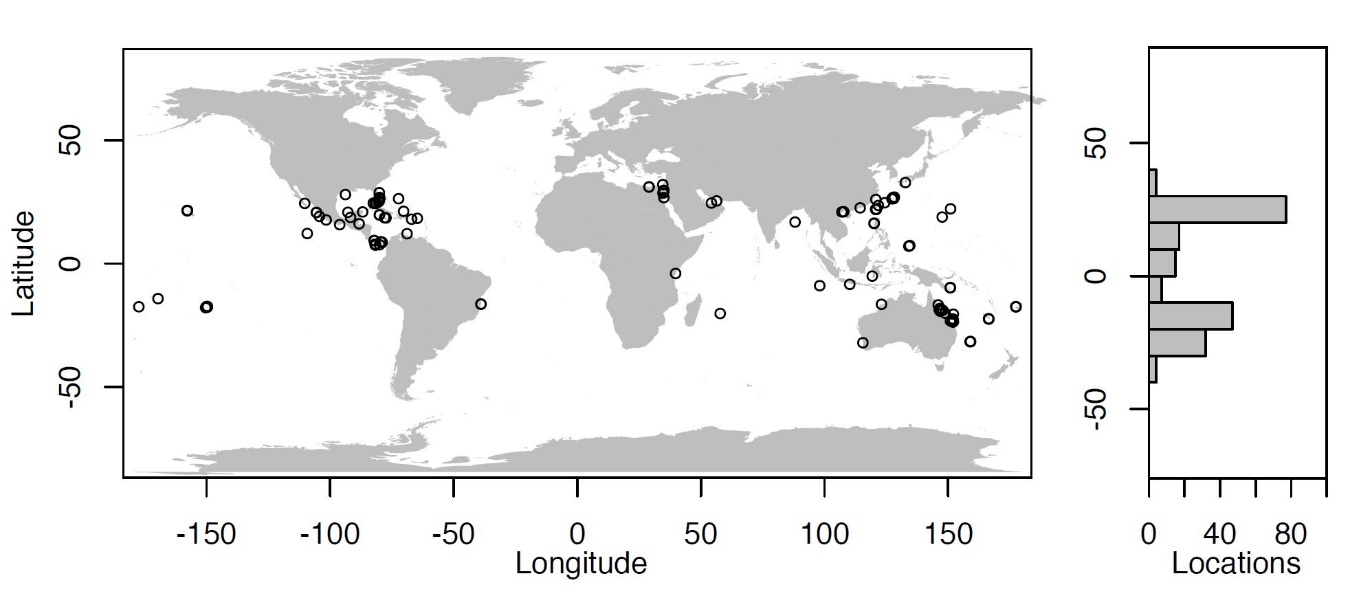
**

**Figure S3. Global location of the study sites (*n* = 172 studies at locations) included in the database and used in the meta-analyses.** The right-hand panel shows the latitudinal distribution of studies (± 35 degrees of latitude).

**
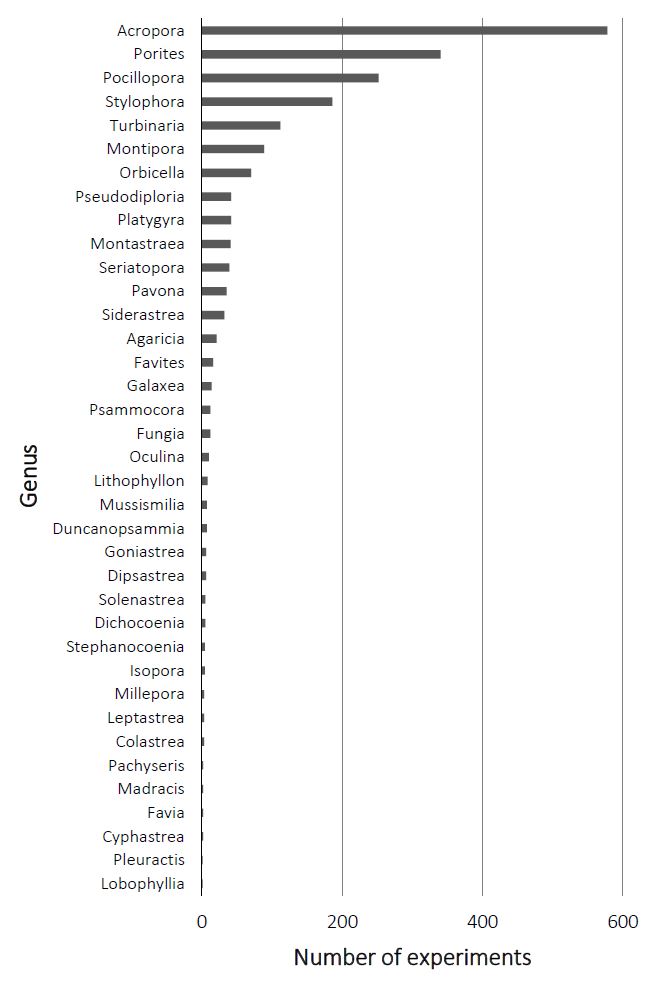
**

**Figure S4. Number of replicated experiments per genus included in the database and used in the meta-analysis.**


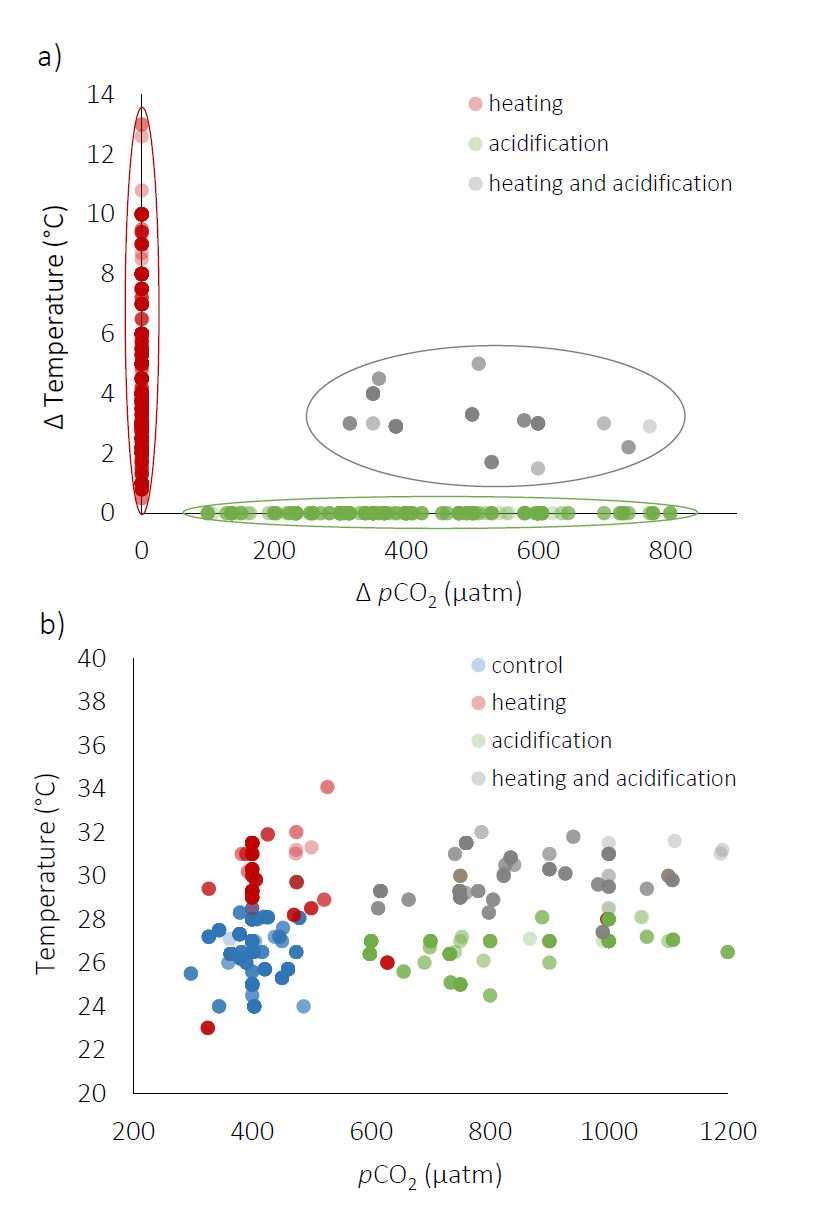


**Figure S5.** A, The relationship between increases in temperature and *p*CO_2_ relative to control conditions in heating, acidification, and heating & acidification treatments. B, The relationship between absolute temperature and *p*CO_2_ concentrations in control and the heated, acidification, and heating & acidification treatments.

Table S1. Simulated changes in the maximum annual intensity (°C) and annual mean duration of MHWs (days) relative to preindustrial times (1861-1880) exceeding the 90^th^ and 99^th^ percentile for different global warming scenarios (cf. methods). The different representative concentration pathways (RCPs) represent a low emission scenario (RCP2.6), an intermediate emission scenario (RCP4.5), and a high emission scenario (RCP8.5).

| **RCPs** | **Period (years)** | **Percentile of SST** | **Global warming (relative to 1861-1880)** | **Global warming (relative to 1991-2010)** | **Maximum Annual Intensity of MHWs** | **Annual mean duration of MHWs** |
| --- | --- | --- | --- | --- | --- | --- |
| **Historical** | 1861–1880 | 90th | 0 | -0.73 | 0.68 | 23.03 |
| **Historical** | 1991-2010 | 90th | 0.73 | 0 | 1.2 | 41.23 |
| **RCP2.6** | 2021-2040 | 90th | 1.47 | 0.74 | 1.8 | 78.36 |
| **RCP2.6** | 2041-2060 | 90th | 1.7 | 0.97 | 1.96 | 88 |
| **RCP2.6** | 2081-2100 | 90th | 1.75 | 1.02 | 2 | 91.23 |
| **RCP4.5** | 2021-2040 | 90th | 1.52 | 0.79 | 1.83 | 80.55 |
| **RCP4.5** | 2041-2060 | 90th | 2.01 | 1.28 | 2.24 | 103.7 |
| **RCP4.5** | 2081-2100 | 90th | 2.55 | 1.82 | 2.69 | 129.3 |
| **RCP8.5** | 2021-2040 | 90th | 1.63 | 0.9 | 1.94 | 86.14 |
| **RCP8.5** | 2041-2060 | 90th | 2.46 | 1.73 | 2.63 | 125 |
| **RCP8.5** | 2081-2100 | 90th | 4.48 | 3.75 | 4.32 | 213.4 |
| **Historical** | 1861–1880 | 99th | 0 | -0.73 | 0.29 | 8.97 |
| **Historical** | 1991-2010 | 99th | 0.73 | 0 | 0.59 | 18.11 |
| **RCP2.6** | 2021-2040 | 99th | 1.47 | 0.74 | 1.02 | 41.4 |
| **RCP2.6** | 2041-2060 | 99th | 1.7 | 0.97 | 1.16 | 49.25 |
| **RCP2.6** | 2081-2100 | 99th | 1.75 | 1.02 | 1.2 | 51.92 |
| **RCP4.5** | 2021-2040 | 99th | 1.52 | 0.79 | 1.05 | 43.91 |
| **RCP4.5** | 2041-2060 | 99th | 2.01 | 1.28 | 1.41 | 63.14 |
| **RCP4.5** | 2081-2100 | 99th | 2.55 | 1.82 | 1.84 | 87.43 |
| **RCP8.5** | 2021-2040 | 99th | 1.63 | 0.9 | 1.15 | 47.98 |
| **RCP8.5** | 2041-2060 | 99th | 2.46 | 1.73 | 1.78 | 82.49 |
| **RCP8.5** | 2081-2100 | 99th | 4.48 | 3.75 | 3.44 | 172 |

Table S2. List of the 87 calcifying coral species from which responses were analysed in this study. Species are listed according to the number of observations included in the dataset. For 83 out of the 1,788 number of experimental observations in this study, taxa were identified to genus or family level (Stylophora spp. [*n* = 3], Porites spp. [*n* = 75], Agaricia spp. [*n* = 4], Pocilloporidae sp. [*n* = 1]).

| GENUS | SPECIES | NUMBER OF OBSERVATIONS |
| --- | --- | --- |
| ***Pocillopora*** | *damicornis* | 174 |
| ***Stylophora*** | *pistillata* | 172 |
| ***Acropora*** | *millepora* | 142 |
| ***Turbinaria*** | *reniformis* | 100 |
| ***Porites*** | *sp.* | 75 |
| ***Porites*** | *astreoides* | 69 |
| ***Acropora*** | *digitifera* | 63 |
| ***Acropora*** | *tenuis* | 61 |
| ***Acropora*** | *hyacinthus* | 57 |
| ***Acropora*** | *muricata* | 54 |
| ***Acropora*** | *pulchra* | 54 |
| ***Pocillopora*** | *verrucosa* | 44 |
| ***Orbicella*** | *faveolata* | 40 |
| ***Montastraea*** | *cavernosa* | 39 |
| ***Porites*** | *rus* | 39 |
| ***Acropora*** | *cervicornis* | 38 |
| ***Montipora*** | *digitata* | 37 |
| ***Pseudodiploria*** | *strigosa* | 34 |
| ***Porites*** | *lobata* | 32 |
| ***Porites*** | *cylindrica* | 31 |
| ***Orbicella*** | *annularis* | 28 |
| ***Acropora*** | *aspera* | 27 |
| ***Acropora*** | *palmata* | 25 |
| ***Montipora*** | *monasteriata* | 25 |
| ***Seriatopora*** | *hystrix* | 24 |
| ***Platygyra*** | *acuta* | 20 |
| ***Acropora*** | *intermedia* | 19 |
| ***Porites*** | *irregularis* | 19 |
| ***Favites*** | *complanata* | 18 |
| ***Pavona*** | *cactus* | 18 |
| ***Platygyra*** | *daedalea* | 18 |
| ***Porites*** | *solida* | 18 |
| ***Pocillopora*** | *capitata* | 15 |
| ***Seriatopora*** | *caliendrum* | 15 |
| ***Siderastrea*** | *siderea* | 15 |
| ***Galaxea*** | *fascicularis* | 14 |
| ***Porites*** | *compressa* | 14 |
| ***Montipora*** | *capitata* | 13 |
| ***Porites*** | *lutea* | 13 |
| ***Siderastrea*** | *radians* | 13 |
| ***Montipora*** | *verrucosa* | 12 |
| ***Turbinaria*** | *mesenterina* | 12 |
| ***Pocillopora*** | *meandrina* | 11 |
| ***Oculina*** | *patagonica* | 10 |
| ***Psammocora*** | *profundacella* | 9 |
| ***Acropora*** | *selago* | 8 |
| ***Platygyra*** | *ryukyuensis* | 8 |
| ***Agaricia*** | *agaricites* | 7 |
| ***Duncanopsammia*** | *axifuga* | 7 |
| ***Mussismilia*** | *harttii* | 7 |
| ***Goniastrea*** | *fascicularis* | 6 |
| ***Pavona*** | *decussata* | 6 |
| ***Porites*** | *australiensis* | 6 |
| ***Porites*** | *porites* | 6 |
| ***Fungia*** | *fungites* | 5 |
| ***Pavona*** | *gigantea* | 5 |
| ***Agaricia*** | *lamarki* | 4 |
| ***Agaricia*** | *sp.* | 4 |
| ***Lithophyllon*** | *repanda* | 4 |
| ***Pavona*** | *clavus* | 4 |
| ***Pocillopora*** | *elegans* | 4 |
| ***Porites*** | *divaricata* | 4 |
| ***Porites*** | *heronensis* | 4 |
| ***Pseudodiploria*** | *clivosa* | 4 |
| ***Stephanocoenia*** | *intersepta* | 4 |
| ***Acropora*** | *yongei* | 3 |
| ***Dichocoenia*** | *stokesii* | 3 |
| ***Fungia*** | *scutaria* | 3 |
| ***Isopora*** | *palifera* | 3 |
| ***Leptastrea*** | *purpurea* | 3 |
| ***Millepora*** | *platyphylla* | 3 |
| ***Solenastrea*** | *hyades* | 3 |
| ***Stylophora*** | *spp.* | 3 |
| ***Acropora*** | *hemprichii* | 2 |
| ***Cyphastrea*** | *serailia* | 2 |
| ***Montipora*** | *aequituberculata* | 2 |
| ***Pachyseris*** | *rugosa* | 2 |
| ***Acroporidae*** | *spp.* | 1 |
| ***Agaricia*** | *tenuifolia* | 1 |
| ***Faviidae*** | *spp.* | 1 |
| ***Isopora*** | *cuneata* | 1 |
| ***Lobophyllia*** | *corymbosa* | 1 |
| ***Madracis*** | *auretenra* | 1 |
| ***Pavona*** | *varians* | 1 |
| ***Pleuractis*** | *granulosa* | 1 |
| ***Pocilloporidae*** | *spp.* | 1 |
| ***Porites*** | *panamensis* | 1 |

Table S3. Number of experimental observations of calcifying coral responses to warming and acidification used in the meta-analysis (total *n* = 1,788). Sample sizes (*n*) for each response variable differ between activation energy (*E,* eV) estimates for warming + acidification and CO_2_ Ln*RR* (Δ 100 µatm^-1^) estimates of acidification + warming due to some studies that did not report *p*CO_2_ in nominal acidification treatments.

| **Biological process** | **Warming (E, eV)** | **Warming + Acidification  (E, eV)** | **Acidification (CO_2_ LnRR Δ 100 µatm^-1^)** | **Acidification + Warming  (CO_2_ LnRR Δ 100 µatm^-1^)** |
| --- | --- | --- | --- | --- |
| **Symbiont density** | 283 | 21 | 20 | 20 |
| **Chla content** | 167 | 22 | 24 | 21 |
| **Photosynthesis** | 73 | 21 | 45 | 14 |
| **Photochemical efficiency** | 323 | 37 | 34 | 33 |
| **Calcification** | 100 | 77 | 202 | 69 |
| **Growth** | 36 | 4 | 9 | 4 |
| **Survival** | 91 | 8 | 5 | 8 |
| **Holobiont Respiration** | 93 | 36 | 57 | 30 |
| **Total observations** | 1,166 | 226 | 396 | 199 |

**Table S4** Summary of results of mixed-effects meta-analyses for each coral process. Estimates of statistical sensitivity for each coral attribute represent the minimum increase in treatment level (temperature in °C or *p*CO_2_ in µatm) required to produce a significant percent change. Statistical sensitivity estimates were derived from mean effect sizes (Activation Energy [*E*, eV] and CO_2_ Ln*RR* ∆100 µatm^-1^) and 95% CI resulting from the meta-analyses. *P*-values in bold represent are significant (*P* < 0.05). Estimates in bold represent statistically significant effects (*P* < 0.05).

| CORAL ATTRIBUTE | EFFECT SIZE | | FACTOR(S) | MEAN ESTIMATE | *P*-VALUE | CI (±95%) | *N* | SENSITIVITY  ESTIMATE | PROCESS CHANGE AT SENSITIVITY ESTIMATE (%) |
| --- | --- | --- | --- | --- | --- | --- | --- | --- | --- |
| *Symbiont density* | | *E* (eV) | Heating | 1.50938 | **0.00012** | 0.76793 | 283 | **< 0.50 °C** | **- 8.3%** |
|  | | *E* (eV) | Heating [+ Acidification] | 0.68194 | **0.00203** | 0.4332 | 21 | **0.57 °C** | **- 5.25%** |
|  | | Ln*RR* ∆100 µatm^-1^ | Acidification | -0.00363 | 0.96693 | 0.1715 | 20 | > 800 µatm |  |
|  | | Ln*RR* ∆100 µatm^-1^ | Acidification [+ Heating] | -0.10546 | 0.30927 | 0.20329 | 20 | **215** **µatm** | **- 20.2%** |
| *Chla content* | | *E* (eV) | Heating | 0.45238 | **0.00737** | 0.33088 | 163 | **0.7 °C** | **- 4.1%** |
|  | | *E* (eV) | Heating [+ Acidification] | 0.477195 | 0.22705 | 0.77424 | 22 | **1.66 °C** | **- 9.8%** |
|  | | Ln*RR* ∆100 µatm^-1^ | Acidification | -0.01605 | 0.82179 | 0.13974 | 24 | > 800 µatm |  |
|  | | Ln*RR* ∆100 µatm^-1^ | Acidification [+ Heating] | -0.04185 | 0.60934 | 0.16052 | 21 | **437 µatm** | **- 16.7%** |
| *Photosynthesis* | | *E* (eV) | Heating | 0.393757 | **0.04762** | 0.389627 | 73 | **0.97 °C** | **- 4.9%** |
|  | | *E* (eV) | Heating [+ Acidification] | 0.930159 | **0.02448** | 0.810462 | 21 | **0.84 °C** | **- 9.8%** |
|  | | Ln*RR* ∆100 µatm^-1^ | Heating | 0.005561 | 0.73124 | 0.118620 | 45 | > 800 µatm |  |
|  | | Ln*RR* ∆100 µatm^-1^ | Acidification [+ Heating] | -0.048603 | 0.80441 | 0.384682 | 14 | > 800 µatm |  |
| *Photochemical* | | *E* (eV) | Heating | 0.259104 | **0.00220** | 0.002593 | 323 | **0.62 ⁰C** | **- 2.1%** |
| *efficiency* | | *E* (eV) | Heating [+ Acidification] | 0.029008 | 0.894334 | 0.428053 | 9 | > 10 ⁰C |  |
|  | | Ln*RR* ∆100 µatm^-1^ | Acidification | -0.000561 | 0.995609 | 0.199710 | 34 | > 800 µatm |  |
|  | | Ln*RR* ∆100 µatm^-1^ | Acidification [+ Heating] | -0.003974 | 0.972463 | 0.225668 | 33 | > 800 µatm |  |
| *Calcification* | | *E* (eV) | Heating | 0.295408 | **0.01412** | 0.235925 | 100 | **0.77 °C** | **- 3%** |
|  | | *E* (eV) | Heating [+ Acidification] | 0.421122 | 0.134247 | 0.551153 | 77 | **1.31 ⁰C** | **- 7%** |
|  | | Ln*RR* ∆100 µatm^-1^ | Acidification | -0.019746 | 0.565856 | 0.067405 | 202 | **359 µatm** | **- 6.8%** |
|  | | Ln*RR* ∆100 µatm^-1^ | Acidification [+ Warming] | -0.028689 | 0.651715 | 0.124572 | 69 | **482 µatm** | **- 12.9%** |
| *Growth* | | *E* (eV) | Heating | -0.007890 | 0.969903 | 0.409871 | 36 | > 10 ⁰C |  |
|  | | *E* (eV) | Heating [+ Acidification] | - | - | - | 4 | - |  |
|  | | Ln*RR* ∆100 µatm^-1^ | Acidification | -0.015288 | 0.898029 | 0.233817 | 9 | > 800 µatm |  |
|  | | Ln*RR* ∆100 µatm^-1^ | Acidification [+ Heating] | - | - | - | 4 | - |  |
| *Survival* | | *E* (eV) | Heating | 0.6642669 | **0.047818** | 0.657883 | 91 | **0.97 ⁰C** | **- 8.2%** |
|  | | *E* (eV) | Warming [+ Acidification] | 2.4054038 | 0.122282 | 3.050941 | 8 | **1.38 °C** | **- 35.3%** |
|  | | Ln*RR* ∆100 µatm^-1^ | Acidification | - | - | - | 5 | - |  |
|  | | Ln*RR* ∆100 µatm^-1^ | Acidification [+ Warming] | -0.1072542 | 0.346058 | 0.223095 | 8 | **238 µatm** | **- 22.5%** |
| *Respiration* | | *E* (eV) | Heating | 0.014765 | 0.913826 | 0.000001 | 93 | > 10 ⁰C |  |
|  | | *E* (eV) | Heating [+ Acidification] | -0.066679 | 0.683703 | 0.000001 | 36 | **4.82 °C** | **+ 4.2%** |
|  | | Ln*RR* ∆100 µatm^-1^ | Acidification | 0.007033 | 0.941579 | 0.188109 | 57 | > 800 µatm |  |
|  | | Ln*RR* ∆100 µatm^-1^ | Acidification [+Heating] | 0.030765 | 0.791123 | 0.227671 | 30 | > 800 µatm |  |

**Table S5.** List of the 172 publications analysed in the meta-analysis.

| Authors | Title | Journal | Volume | Year |
| --- | --- | --- | --- | --- |
| Baria. MVB; Kurihara. H; Harii. S | Tolerance to Elevated Temperature and Ocean Acidification of the Larvae of the Solitary Corals *Fungia fungites* (Linnaues. 1758) and *Lithophyllon repanda* (Dana. 1846) | ZOOLOGICAL SCIENCE | 32 | 2015 |
| Dufault. AM; Ninokawa. A; Bramanti. L; Cumbo. VR; Fan. TY; Edmunds. PJ | The role of light in mediating the effects of ocean acidification on coral calcification | JOURNAL OF EXPERIMENTAL BIOLOGY | 63 | 2013 |
| Schoepf. V; Grottoli. AG; Warner. ME; Cai. WJ; Melman. TF; Hoadley. KD; Pettay. DT; Hu. XP; Li. Q; Xu. H; Wang. YC; Matsui. Y; Baumann. JH | Coral Energy Reserves and Calcification in a High-CO_2_ World at Two Temperatures | PLOS ONE | 8 | 2013 |
| Biscere. T; Lorrain. A; Rodolfo-Metalpa. R; Gilbert. A; Wright. A; Devissi. C; Peignon. C; Farman. R; Duvieilbourg. E; Payri. C; Houlbreque. F | Nickel and ocean warming affect scleractinian coral growth | MARINE POLLUTION BULLETIN | 120 | 2017 |
| Hoadley. KD; Pettay. DT; Grottoli. AG; Cai. WJ; Melman. TF; Schoepf. V; Hu. XP; Li. Q; Xu. H; Wang. YC; Matsui. Y; Baumann. JH; Warner. ME | Physiological response to elevated temperature and *p*CO_2_ varies across four Pacific coral species: Understanding the unique host plus symbiont response | SCIENTIFIC REPORTS | 5 | 2015 |
| Kavousi. J; Reimer. JD; Tanaka. Y; Nakamura. T | Colony-specific investigations reveal highly variable responses among individual corals to ocean acidification and warming | MARINE ENVIRONMENTAL RESEARCH | 109 | 2015 |
| Bellantuono. AJ; Hoegh-Guldberg. O; Rodriguez-Lanetty. M | Resistance to thermal stress in corals without changes in symbiont composition | PROCEEDINGS OF THE ROYAL SOCIETY B-BIOLOGICAL SCIENCES | 83 | 2012 |
| Olsen. K; Ritson-Williams. R; Ochrietor. JD; Paul. VJ; Ross. C | Detecting hyperthermal stress in larvae of the hermatypic coral *Porites astreoides*: the suitability of using biomarkers of oxidative stress versus heat-shock protein transcriptional expression | MARINE BIOLOGY | 160 | 2013 |
| Middlebrook. R; Anthony. KRN; Hoegh-Guldberg. O; Dove. S | Heating rate and symbiont productivity are key factors determining thermal stress in the reef-building coral *Acropora formosa* | JOURNAL OF EXPERIMENTAL BIOLOGY | 60 | 2010 |
| Ritson-Williams. R; Ross. C; Paul. VJ | Elevated Temperature and Allelopathy Impact Coral Recruitment | PLOS ONE | 11 | 2016 |
| Hawkins. TD; Krueger. T; Wilkinson. SP; Fisher. PL; Davy. SK | Antioxidant responses to heat and light stress differ with habitat in a common reef coral | CORAL REEFS | 34 | 2015 |
| Pontasch. S; Fisher. PL; Krueger. T; Dove. S; Hoegh-Guldberg. O; Leggat. W; Davy. SK | Photoacclimatory and photo protective responses to cold versus heat stress in high latitude reef corals | JOURNAL OF PHYCOLOGY | 53 | 2017 |
| Ross. C; Ritson-Williams. R; Olsen. K; Paul. VJ | Short-term and latent post-settlement effects associated with elevated temperature and oxidative stress on larvae from the coral *Porites astreoides* | CORAL REEFS | 101 | 2013 |
| Comeau. S; Carpenter. RC; Edmunds. PJ | Effects of feeding and light intensity on the response of the coral Porites rus to ocean acidification | MARINE BIOLOGY | 45 | 2013 |
| Anthony. KRN; Kline. DI; Diaz-Pulido. G; Dove. S; Hoegh-Guldberg. O | Ocean acidification causes bleaching and productivity loss in coral reef builders | PROCEEDINGS OF THE NATIONAL ACADEMY OF SCIENCES OF THE UNITED STATES OF AMERICA | 6 | 2008 |
| McGinley. MP; Aschaffenburg. MD; Pettay. DT; Smith. RT; LaJeunesse. TC; Warner. ME | Transcriptional Response of Two Core Photosystem Genes in Symbiodinium spp. Exposed to Thermal Stress | PLOS ONE | 173 | 2012 |
| Bhagooli. R; Hidaka. M | Photoinhibition. bleaching susceptibility and mortality in two scleractinian corals. *Platygyra ryukyuensis* and *Stylophora pistillata*. in response to thermal and light stresses | COMPARATIVE BIOCHEMISTRY AND PHYSIOLOGY A-MOLECULAR & INTEGRATIVE PHYSIOLOGY | 6 | 2004 |
| Bassim. KM; Sammarco. PW | Effects of temperature and ammonium on larval development and survivorship in a scleractinian coral (*Diploria strigosa*) | MARINE BIOLOGY | 10 | 2003 |
| Smith-Keune. C; Dove. S | Gene expression of a green fluorescent protein homolog as a host-specific biomarker of heat stress within a reef-building coral | MARINE BIOTECHNOLOGY | 3 | 2008 |
| Edmunds. PJ | The effect of sub-lethal increases in temperature on the growth and population trajectories of three scleractinian corals on the southern Great Barrier Reef | OECOLOGIA | 31 | 2005 |
| Lee. STM; Davy. SK; Tang. SL; Kench. PS | Mucus Sugar Content Shapes the Bacterial Community Structure in Thermally Stressed *Acropora muricata* | FRONTIERS IN MICROBIOLOGY | 7 | 2016 |
| Ezzat. L; Towle. E; Irisson. JO; Langdon. C; Ferrier-Pages. C | The relationship between heterotrophic feeding and inorganic nutrient availability in the scleractinian coral *T-reniformis* under a short-term temperature increase | LIMNOLOGY AND OCEANOGRAPHY | 61 | 2016 |
| Comeau. S; Carpenter. RC; Edmunds. PJ | Effects of pCO_2_ on photosynthesis and respiration of tropical scleractinian corals and calcified algae | ICES JOURNAL OF MARINE SCIENCE | 74 | 2017 |
| Fujise. L; Yamashita. H; Suzuki. G; Sasaki. K; Liao. LM; Koike. K | Moderate Thermal Stress Causes Active and Immediate Expulsion of Photosynthetically Damaged Zooxanthellae (Symbiodinium) from Corals | PLOS ONE | 9 | 2014 |
| Strychar. KB; Coates. M; Sammarco. PW | Loss of Symbiodinium from bleached Australian scleractinian corals (*Acropora hyacinthus. Favites complanata and Porites solida*) | MARINE AND FRESHWATER RESEARCH | 56 | 2004 |
| Ferrier-Pages. C; Rottier. C; Beraud. E; Levy. O | Experimental assessment of the feeding effort of three scleractinian coral species during a thermal stress: Effect on the rates of photosynthesis | JOURNAL OF EXPERIMENTAL MARINE BIOLOGY AND ECOLOGY | 127 | 2010 |
| Iguchi. A; Kumagai. NH; Nakamura. T; Suzuki. A; Sakai. K; Nojiri. Y | Responses of calcification of massive and encrusting corals to past, present, and near-future ocean carbon dioxide concentrations | MARINE POLLUTION BULLETIN | 89 | 2014 |
| Renegar. DA; Riegl. BM | Effect of nutrient enrichment and elevated CO_2_ partial pressure on growth rate of Atlantic scleractinian coral *Acropora cervicornis* | MARINE ECOLOGY PROGRESS SERIES | 89 | 2005 |
| Kushmaro. A; Rosenberg. E; Fine. M; Ben Haim. Y; Loya. Y | Effect of temperature on bleaching of the coral *Oculina patagonica* by Vibrio AK-1 | MARINE ECOLOGY PROGRESS SERIES | 14 | 1998 |
| Reynaud. S; Leclercq. N; Romaine-Lioud. S; Ferrier-Pages. C; Jaubert. J; Gattuso. JP | Interacting effects of CO_2_ partial pressure and temperature on photosynthesis and calcification in a scleractinian coral | GLOBAL CHANGE BIOLOGY | 71 | 2003 |
| van Dam. JW; Uthicke. S; Beltran. VH; Mueller. JF; Negri. AP | Combined thermal and herbicide stress in functionally diverse coral symbionts | ENVIRONMENTAL POLLUTION | 204 | 2015 |
| Medellin-Maldonado. F; Cabral-Tena. RA; Lopez-Perez. A; Calderon-Aguilera. LE; Norzagaray-Lopez. CO; Chapa-Balcorta. C; Zepeta-Vilchis. RC | Calcification of the main reef-building coral species on the Pacific coast of southern Mexico | CIENCIAS MARINAS | 42 | 2016 |
| Anderson. KD; Heron. SF; Pratchett. MS | Species-specific declines in the linear extension of branching corals at a subtropical reef. Lord Howe Island | CORAL REEFS | 34 | 2015 |
| Brown. D; Edmunds. PJ | Differences in the responses of three scleractinians and the hydrocoral *Millepora platyphylla* to ocean acidification | MARINE BIOLOGY | 163 | 2016 |
| Edmunds. PJ; Burgess. SC | Size-dependent physiological responses of the branching coral *Pocillopora verrucosa* to elevated temperature and *p*CO_2_ | JOURNAL OF EXPERIMENTAL BIOLOGY | 219 | 2016 |
| Abrego. D; Ulstrup. KE; Willis. BL; van Oppen. MJH | Species-specific interactions between algal endosymbionts and coral hosts define their bleaching response to heat and light stress | PROCEEDINGS OF THE ROYAL SOCIETY B-BIOLOGICAL SCIENCES | 81 | 2008 |
| Wall. CB; Fan. TY; Edmunds. PJ | Ocean acidification has no effect on thermal bleaching in the coral *Seriatopora caliendrum* | CORAL REEFS | 33 | 2014 |
| Takahashi. A; Kurihara. H | Ocean acidification does not affect the physiology of the tropical coral *Acropora digitifera* during a 5-week experiment | CORAL REEFS | 101 | 2013 |
| Rodriguez-Lanetty. M; Harii. S; Hoegh-Guldberg. O | Early molecular responses of coral larvae to hyperthermal stress | MOLECULAR ECOLOGY | 50 | 2009 |
| Wicks. LC; Hill. R; Davy. SK | The influence of irradiance on tolerance to high and low temperature stress exhibited by Symbiodinium in the coral. *Pocillopora damicornis.* from the high-latitude reef of Lord Howe Island | LIMNOLOGY AND OCEANOGRAPHY | 162 | 2010 |
| Winter. APM; Chaloub. RM; Duarte. GAS; Castro. CBE | Photosynthetic responses of corals *Mussismilia harttii* (Verrill. 1867) from turbid waters to changes in temperature and presence/absence of light | BRAZILIAN JOURNAL OF OCEANOGRAPHY | 64 | 2016 |
| Yuyama. I; Nakamura. T; Higuchi. T; Hidaka. M | Different Stress Tolerances of Juveniles of the Coral *Acropora tenuis* Associated with Clades C1 and D Symbiodinium | ZOOLOGICAL STUDIES | 55 | 2016 |
| Hawkins. TD; Krueger. T; Becker. S; Fisher. PL; Davy. SK | Differential nitric oxide synthesis and host apoptotic events correlate with bleaching susceptibility in reef corals | CORAL REEFS | 33 | 2014 |
| Jury. CP; Whitehead. RF; Szmant. AM | Effects of variations in carbonate chemistry on the calcification rates of *Madracis auretenra* (= *Madracis mirabilis* s*ensu* Wells. 1973): bicarbonate concentrations best predict calcification rates | GLOBAL CHANGE BIOLOGY | 44 | 2010 |
| Gilbert. JA; Hill. R; Doblin. MA; Ralph. PJ | Microbial consortia increase thermal tolerance of corals | MARINE BIOLOGY | 43 | 2012 |
| Randall. CJ; Szmant. AM | Elevated Temperature Affects Development. Survivorship. and Settlement of the Elkhorn Coral. *Acropora palmata* (Lamarck 1816) | BIOLOGICAL BULLETIN | 64 | 2009 |
| Negri. AP; Flores. F; Rothig. T; Uthicke. S | Herbicides increase the vulnerability of corals to rising sea surface temperature | LIMNOLOGY AND OCEANOGRAPHY | 163 | 2011 |
| Abrego. D; Willis. BL; van Oppen. MJH | Impact of Light and Temperature on the Uptake of Algal Symbionts by Coral Juveniles | PLOS ONE | 173 | 2012 |
| Putnam. HM; Gates. RD | Preconditioning in the reef-building coral Pocillopora damicornis and the potential for trans-generational acclimatization in coral larvae under future climate change conditions | JOURNAL OF EXPERIMENTAL BIOLOGY | 218 | 2015 |
| Silverstein. RN; Cunning. R; Baker. AC | Tenacious D: Symbiodinium in clade D remain in reef corals at both high and low temperature extremes despite impairment | JOURNAL OF EXPERIMENTAL BIOLOGY | 220 | 2017 |
| Inoue. M; Shinmen. K; Kawahata. H; Nakamura. T; Tanaka. Y; Kato. A; Shinzato. C; Iguchi. A; Kan. H; Suzuki. A; Sakai. K | Estimate of calcification responses to thermal and freshening stresses based on culture experiments with symbiotic and aposymbiotic primary polyps of a coral. *Acropora digitifera* | GLOBAL AND PLANETARY CHANGE | 192 | 2012 |
| Chui. APY; Ang. P | High tolerance to temperature and salinity change should enable scleractinian coral *Platygyra acuta* from marginal environments to persist under future climate change | PLOS ONE | 12 | 2017 |
| Hill. R; Takahashi. S | Photosystem II recovery in the presence and absence of chloroplast protein repair in the symbionts of corals exposed to bleaching conditions | CORAL REEFS | 33 | 2014 |
| Edmunds. PJ | Differential effects of high temperature on the respiration of juvenile Caribbean corals | BULLETIN OF MARINE SCIENCE | 183 | 2008 |
| Sorek. M; Levy. O | The effect of temperature compensation on the circadian rhythmicity of photosynthesis in Symbiodinium. coral-symbiotic alga | SCIENTIFIC REPORTS | 52 | 2012 |
| Ainsworth. TD; Heron. SF; Ortiz. JC; Mumby. PJ; Grech. A; Ogawa. D; Eakin. CM; Leggat. W | Climate change disables coral bleaching protection on the Great Barrier Reef | SCIENCE | 352 | 2016 |
| Haryanti. D; Yasuda. N; Harii. S; Hidaka. M | High tolerance of symbiotic larvae of Pocillopora damicornis to thermal stress | ZOOLOGICAL STUDIES | 54 | 2015 |
| Gierz. SL; Gordon. BR; Leggat. W | Integral Light-Harvesting Complex Expression In Symbiodinium Within The Coral *Acropora aspera* Under Thermal Stress | SCIENTIFIC REPORTS | 6 | 2016 |
| Bhagooli. R; Hidaka. M | Release of zooxanthellae with intact photosynthetic activity by the coral *Galaxea fascicularis* in response to high temperature stress | MARINE BIOLOGY | 30 | 2004 |
| DeSalvo. MK; Sunagawa. S; Voolstra. CR; Medina. M | Transcriptomic responses to heat stress and bleaching in the elkhorn coral *Acropora palmata* | MARINE ECOLOGY PROGRESS SERIES | 134 | 2010 |
| Comeau. S; Edmunds. PJ; Spindel. NB; Carpenter. RC | Fast coral reef calcifiers are more sensitive to ocean acidification in short-term laboratory incubations | LIMNOLOGY AND OCEANOGRAPHY | 59 | 2014 |
| Cumbo. VR; Fan. TY; Edmunds. PJ | Effects of exposure duration on the response of *Pocillopora damicornis* larvae to elevated temperature and high *p*CO_2_ | JOURNAL OF EXPERIMENTAL MARINE BIOLOGY AND ECOLOGY | 142 | 2013 |
| Maor-Landaw. K; Ben-Asher. HW; Karako-Lampert. S; Salmon-Divon. M; Prada. F; Caroselli. E; Goffredo. S; Falini. G; Dubinsky. Z; Levy. O | Mediterranean versus Red sea corals facing climate change. a transcriptome analysis | SCIENTIFIC REPORTS | 7 | 2017 |
| Biscere. T; Rodolfo-Metalpa. R; Lorrain. A; Chauvaud. L; Thebault. J; Clavier. J; Houlbreque. F | Responses of Two Scleractinian Corals to Cobalt Pollution and Ocean Acidification | PLOS ONE | 10 | 2015 |
| Jimenez. IM; Larkum. AWD; Ralph. PJ; Kuhl. M | Thermal effects of tissue optics in symbiont-bearing reef-building corals | LIMNOLOGY AND OCEANOGRAPHY | 164 | 2012 |
| Edmunds. PJ | Zooplanktivory ameliorates the effects of ocean acidification on the reef coral *Porites* spp. | LIMNOLOGY AND OCEANOGRAPHY | 163 | 2011 |
| Silverstein. RN; Cunning. R; Baker. AC | Change in algal symbiont communities after bleaching. not prior heat exposure. increases heat tolerance of reef corals | GLOBAL CHANGE BIOLOGY | 21 | 2015 |
| Fournie. JW; Vivian. DN; Yee. SH; Courtney. LA; Barron. MG | Comparative sensitivity of six scleractinian corals to temperature and solar radiation | DISEASES OF AQUATIC ORGANISMS | 195 | 2012 |
| Kenkel. CD; Goodbody-Gringley. G; Caillaud. D; Davies. SW; Bartels. E; Matz. MV | Evidence for a host role in thermotolerance divergence between populations of the mustard hill coral (*Porites astreoides*) from different reef environments | MOLECULAR ECOLOGY | 22 | 2013 |
| Kaniewska. P; Chan. CKK; Kline. D; Ling. EYS; Rosic. N; Edwards. D; Hoegh-Guldberg. O; Dove. S | Transcriptomic Changes in Coral Holobionts Provide Insights into Physiological Challenges of Future Climate and Ocean Change | PLOS ONE | 10 | 2015 |
| Cardini. U; van Hoytema. N; Bednarz. VN; Rix. L; Foster. RA; Al-Rshaidat. MMD; Wild. C | Microbial dinitrogen fixation in coral holobionts exposed to thermal stress and bleaching | ENVIRONMENTAL MICROBIOLOGY | 18 | 2016 |
| Vogel. N; Meyer. FW; Wild. C; Uthicke. S | Decreased light availability can amplify negative impacts of ocean acidification on calcifying coral reef organisms | MARINE ECOLOGY PROGRESS SERIES | 521 | 2015 |
| Webster. NS; Uthicke. S; Botte. ES; Flores. F; Negri. AP | Ocean acidification reduces induction of coral settlement by crustose coralline algae | GLOBAL CHANGE BIOLOGY | 51 | 2013 |
| Towle. EK; Enochs. IC; Langdon. C | Threatened Caribbean Coral Is Able to Mitigate the Adverse Effects of Ocean Acidification on Calcification by Increasing Feeding Rate (vol 10. e0123394. 2015) | PLOS ONE | 10 | 2015 |
| Franklin. DJ; Cedres. CMM; Hoegh-Guldberg. O | Increased mortality and photoinhibition in the symbiotic dinoflagellates of the Indo-Pacific coral *Stylophora pistillata* (Esper) after summer bleaching | MARINE BIOLOGY | 33 | 2006 |
| Edmunds. PJ | Effect of acclimatization to low temperature and reduced light on the response of reef corals to elevated temperature | MARINE BIOLOGY | 41 | 2009 |
| Tanaka. Y; Iguchi. A; Nishida. K; Inoue. M; Nakamura. T; Suzuki. A; Sakai. K | Nutrient availability affects the response of juvenile corals and the endosymbionts to ocean acidification | LIMNOLOGY AND OCEANOGRAPHY | 59 | 2014 |
| Schoepf. V; Stat. M; Falter. JL; McCulloch. MT | Limits to the thermal tolerance of corals adapted to a highly fluctuating. naturally extreme temperature environment | SCIENTIFIC REPORTS | 5 | 2015 |
| Strahl. J; Francis. DS; Doyle. J; Humphrey. C; Fabricius. KE | Biochemical responses to ocean acidification contrast between tropical corals with high and low abundances at volcanic carbon dioxide seeps | ICES JOURNAL OF MARINE SCIENCE | 73 | 2016 |
| Higuchi. T; Agostini. S; Casareto. BE; Yoshinaga. K; Suzuki. T; Nakano. Y; Fujimura. H; Suzuki. Y | Bacterial enhancement of bleaching and physiological impacts on the coral *Montipora digitata* | JOURNAL OF EXPERIMENTAL MARINE BIOLOGY AND ECOLOGY | 144 | 2013 |
| Noonan. SHC; Fabricius. KE | Ocean acidification affects productivity but not the severity of thermal bleaching in some tropical corals | ICES JOURNAL OF MARINE SCIENCE | 73 | 2016 |
| Santos. HF; Carmo. FL; Duarte. G; Dini-Andreote. F; Castro. CB; Rosado. AS; van Elsas. JD; Peixoto. RS | Climate change affects key nitrogen-fixing bacterial populations on coral reefs | ISME JOURNAL | 8 | 2014 |
| Edmunds. PJ; Gates. RD | Size-dependent differences in the photophysiology of the reef coral *Porites astreoides* | BIOLOGICAL BULLETIN | 54 | 2004 |
| Nakamura. T; Iguchi. A; Suzuki. A; Sakai. K; Nojiri. Y | Effects of acidified seawater on calcification. photosynthetic efficiencies and the recovery processes from strong light exposure in the coral *Stylophora pistillata* | MARINE ECOLOGY-AN EVOLUTIONARY PERSPECTIVE | 38 | 2017 |
| Desalvo. MK; Voolstra. CR; Sunagawa. S; Schwarz. JA; Stillman. JH; Coffroth. MA; Szmant. AM; Medina. M | Differential gene expression during thermal stress and bleaching in the Caribbean coral *Montastraea faveolata* | MOLECULAR ECOLOGY | 47 | 2008 |
| Lutz. A; Raina. JB; Motti. CA; Miller. DJ; van Oppen. MJH | Host Coenzyme Q Redox State Is an Early Biomarker of Thermal Stress in the Coral *Acropora millepora* | PLOS ONE | 10 | 2015 |
| Deschaseaux. ESM; Jones. GB; Deseo. MA; Shepherd. KM; Kiene. RP; Swan. HB; Harrison. PL; Eyre. BD | Effects of environmental factors on dimethylated sulfur compounds and their potential role in the antioxidant system of the coral holobiont | LIMNOLOGY AND OCEANOGRAPHY | 59 | 2014 |
| Borell. EM; Bischof. K | Feeding sustains photosynthetic quantum yield of a scleractinian coral during thermal stress | OECOLOGIA | 42 | 2008 |
| Evensen. NR; Edmunds. PJ | Interactive effects of ocean acidification and neighboring corals on the growth of *Pocillopora verrucosa* | MARINE BIOLOGY | 163 | 2016 |
| Comeau. S; Cornwall. CE; McCulloch. MT | Decoupling between the response of coral calcifying fluid pH and calcification to ocean acidification | SCIENTIFIC REPORTS | 7 | 2017 |
| Nordemar. I; Nystrom. M; Dizon. R | Effects of elevated seawater temperature and nitrate enrichment on the branching coral *Porites cylindrica* in the absence of particulate food | MARINE BIOLOGY | 10 | 2003 |
| Comeau. S; Carpenter. RC; Edmunds. PJ | Coral reef calcifiers buffer their response to ocean acidification using both bicarbonate and carbonate | PROCEEDINGS OF THE ROYAL SOCIETY B-BIOLOGICAL SCIENCES | 85 | 2013 |
| Edmunds. PJ | Is acclimation beneficial to scleractinian corals. Porites spp.? | MARINE BIOLOGY | 161 | 2014 |
| Levas. S; Grottoli. AG; Warner. ME; Cai. WJ; Bauer. J; Schoepf. V; Baumann. JH; Matsui. Y; Gearing. C; Melman. TF; Hoadley. KD; Pettay. DT; Hu. XP; Li. Q; Xu. H; Wang. YC | Organic carbon fluxes mediated by corals at elevated *p*CO_2_ and temperature | MARINE ECOLOGY PROGRESS SERIES | 519 | 2015 |
| Towle. EK; Enochs. IC; Langdon. C | Threatened Caribbean Coral Is Able to Mitigate the Adverse Effects of Ocean Acidification on Calcification by Increasing Feeding Rate | PLOS ONE | 10 | 2015 |
| Scheufen. T; Kramer. WE; Iglesias-Prieto. R; Enriquez. S | Seasonal variation modulates coral sensibility to heat-stress and explains annual changes in coral productivity | SCIENTIFIC REPORTS | 7 | 2017 |
| Cunning. R; Silverstein. RN; Baker. AC | Investigating the causes and consequences of symbiont shuffling in a multi-partner reef coral symbiosis under environmental change | PROCEEDINGS OF THE ROYAL SOCIETY B-BIOLOGICAL SCIENCES | 282 | 2015 |
| Venn. AA; Tambutte. E; Holcomb. M; Laurent. J; Allemand. D; Tambutte. S | Impact of seawater acidification on pH at the tissue-skeleton interface and calcification in reef corals | PROCEEDINGS OF THE NATIONAL ACADEMY OF SCIENCES OF THE UNITED STATES OF AMERICA | 12 | 2013 |
| Lenz. EA; Edmunds. PJ | Branches and plates of the morphologically plastic coral *Porites rus* are insensitive to ocean acidification and warming | JOURNAL OF EXPERIMENTAL MARINE BIOLOGY AND ECOLOGY | 486 | 2017 |
| Howells. EJ; Abrego. D; Meyer. E; Kirk. NL; Burt. JA | Host adaptation and unexpected symbiont partners enable reef-building corals to tolerate extreme temperatures | GLOBAL CHANGE BIOLOGY | 22 | 2016 |
| Marubini. F; Barnett. H; Langdon. C; Atkinson. MJ | Dependence of calcification on light and carbonate ion concentration for the hermatypic coral *Porites compressa* | MARINE ECOLOGY PROGRESS SERIES | 25 | 2001 |
| Schloder. C; D'Croz. L | Responses of massive and branching coral species to the combined effects of water temperature and nitrate enrichment | JOURNAL OF EXPERIMENTAL MARINE BIOLOGY AND ECOLOGY | 98 | 2004 |
| Agostini. S; Fujimura. H; Higuchi. T; Yuyama. I; Casareto. BE; Suzuki. Y; Nakano. Y | The effects of thermal and high-CO_2_ stresses on the metabolism and surrounding microenvironment of the coral *Galaxea fascicularis* | COMPTES RENDUS BIOLOGIES | 336 | 2013 |
| Rodriguez-Troncoso. AP; Carpizo-Ituarte. E; Cupul-Magana. AL | Differential response to cold and warm water conditions in Pocillopora colonies from the Central Mexican Pacific | JOURNAL OF EXPERIMENTAL MARINE BIOLOGY AND ECOLOGY | 128 | 2010 |
| Imbs. AB; Yakovleva. IM | Dynamics of lipid and fatty acid composition of shallow-water corals under thermal stress: an experimental approach | CORAL REEFS | 97 | 2012 |
| Tremblay. P; Gori. A; Maguer. JF; Hoogenboom. M; Ferrier-Pages. C | Heterotrophy promotes the re-establishment of photosynthate translocation in a symbiotic coral after heat stress | Scientific Reports | 6 | 2016 |
| Middlebrook. R; Hoegh-Guldberg. O; Leggat. W | The effect of thermal history on the susceptibility of reef-building corals to thermal stress | JOURNAL OF EXPERIMENTAL BIOLOGY | 58 | 2008 |
| Krueger. T; Horwitz. N; Bodin. J; Giovani. ME; Escrig. S; Meibom. A; Fine. M | Common reef-building coral in the Northern Red Sea resistant to elevated temperature and acidification | ROYAL SOCIETY OPEN SCIENCE | 4 | 2017 |
| Bahr. KD; Jokiel. PL; Rodgers. KS | Relative sensitivity of five Hawaiian coral species to high temperature under high-*p*CO_2_ conditions | CORAL REEFS | 35 | 2016 |
| Marubini. F; Ferrier-Pages. C; Furla. P; Allemand. D | Coral calcification responds to seawater acidification: a working hypothesis towards a physiological mechanism. | CORAL REEFS | 79 | 2008 |
| D'Croz. L; Mate. JL | Experimental responses to elevated water temperature in genotypes of the reef coral Pocillopora damicornis from upwelling and non-upwelling environments in Panama | CORAL REEFS | 70 | 2004 |
| Jones. A; Berkelmans. R | Potential Costs of Acclimatization to a Warmer Climate: Growth of a Reef Coral with Heat Tolerant vs. Sensitive Symbiont Types | PLOS ONE | 156 | 2010 |
| Krueger. T; Hawkins. TD; Becker. S; Pontasch. S; Dove. S; Hoegh-Guldberg. O; Leggat. W; Fisher. PL; Davy. SK | Differential coral bleaching-Contrasting the activity and response of enzymatic antioxidants in symbiotic partners under thermal stress | COMPARATIVE BIOCHEMISTRY AND PHYSIOLOGY A-MOLECULAR & INTEGRATIVE PHYSIOLOGY | 190 | 2015 |
| Winkler. NS; Pandolfi. JM; Sampayo. EM | Symbiodinium identity alters the temperature-dependent settlement behaviour of *Acropora millepora* coral larvae before the onset of symbiosis | PROCEEDINGS OF THE ROYAL SOCIETY B-BIOLOGICAL SCIENCES | 282 | 2015 |
| Putnam. HM; Edmunds. PJ; Fan. TY | Effect of Temperature on the Settlement Choice and Photophysiology of Larvae From the Reef Coral Stylophora pistillata | BIOLOGICAL BULLETIN | 62 | 2008 |
| Shaw. EC; Carpenter. RC; Lantz. CA; Edmunds. PJ | Intraspecific variability in the response to ocean warming and acidification in the scleractinian coral *Acropora pulchra* | MARINE BIOLOGY | 163 | 2016 |
| Faxneld. S; Jorgensen. TL; Nguyen. ND; Nystrom. M; Tedengren. M | Differences in physiological response to increased seawater temperature in nearshore and offshore corals in northern Vietnam | MARINE ENVIRONMENTAL RESEARCH | 175 | 2011 |
| Visram. S; Douglas. AE | Resilience and acclimation to bleaching stressors in the scleractinian coral *Porites cylindrica* | JOURNAL OF EXPERIMENTAL MARINE BIOLOGY AND ECOLOGY | 112 | 2007 |
| Comeau. S; Edmunds. PJ; Spindel. NB; Carpenter. RC | The responses of eight coral reef calcifiers to increasing partial pressure of CO_2_ do not exhibit a tipping point | LIMNOLOGY AND OCEANOGRAPHY | 165 | 2013 |
| Ainsworth. TD; Hoegh-Guldberg. O; Heron. SF; Skirving. WJ; Leggat. W | Early cellular changes are indicators of pre-bleaching thermal stress in the coral host | JOURNAL OF EXPERIMENTAL MARINE BIOLOGY AND ECOLOGY | 116 | 2008 |
| Kavousi. J; Parkinson. JE; Nakamura. T | Combined ocean acidification and low temperature stressors cause coral mortality | CORAL REEFS | 35 | 2016 |
| Oliver. TA; Palumbi. SR | Do fluctuating temperature environments elevate coral thermal tolerance? | CORAL REEFS | 93 | 2011 |
| Ganase. A; Bongaerts. P; Visser. PM; Dove. SG | The effect of seasonal temperature extremes on sediment rejection in three scleractinian coral species | CORAL REEFS | 35 | 2016 |
| Rodriguez-Troncoso. AP; Carpizo-Ituarte. E; Cupul-Magana. AL | Physiological response to high temperature in the Tropical Eastern Pacific coral *Pocillopora verrucosa* | MARINE ECOLOGY-AN EVOLUTIONARY PERSPECTIVE | 37 | 2016 |
| D'Croz. L; Mate. JL; Oke. JE | Responses to elevated sea water temperature and UV radiation in the coral *Porites lobata* from upwelling and non-upwelling environments on the Pacific coast of Panama | BULLETIN OF MARINE SCIENCE | 62 | 2001 |
| Towle. EK; Baker. AC; Langdon. C | Preconditioning to high CO_2_ exacerbates the response of the Caribbean branching coral *Porites porites* to high temperature stress | MARINE ECOLOGY PROGRESS SERIES | 546 | 2016 |
| Humanes. A; Ricardo. GF; Willis. BL; Fabricius. KE; Negri. AP | Cumulative effects of suspended sediments. organic nutrients and temperature stress on early life history stages of the coral *Acropora tenuis* | SCIENTIFIC REPORTS | 7 | 2017 |
| Courtial. L; Ferrier-Pages. C; Jacquet. S; Rodolfo-Metalpa. R; Reynaud. S; Rottier. C; Houlbreque. F | Effects of temperature and UVR on organic matter fluxes and the metabolic activity of *Acropora muricata* | BIOLOGY OPEN | 6 | 2017 |
| Flores-Ramirez. LA; Linan-Cabello. MA | Relationships among thermal stress. bleaching and oxidative damage in the hermatypic coral. *Pocillopora capitata* | COMPARATIVE BIOCHEMISTRY AND PHYSIOLOGY C-TOXICOLOGY & PHARMACOLOGY | 31 | 2007 |
| Barkley. HC; Cohen. AL; McCorkle. DC; Golbuu. Y | Mechanisms and thresholds for pH tolerance in Palau corals | JOURNAL OF EXPERIMENTAL MARINE BIOLOGY AND ECOLOGY | 489 | 2017 |
| Comeau. S; Carpenter. RC; Edmunds. PJ | Effects of irradiance on the response of the coral *Acropora pulchra* and the calcifying alga *Hydrolithon reinboldii* to temperature elevation and ocean acidification | JOURNAL OF EXPERIMENTAL MARINE BIOLOGY AND ECOLOGY | 453 | 2014 |
| Buerger. P; Schmidt. GM; Wall. M; Held. C; Richter. C | Temperature tolerance of the coral *Porites lutea* exposed to simulated large amplitude internal waves (LAIW) | JOURNAL OF EXPERIMENTAL MARINE BIOLOGY AND ECOLOGY | 471 | 2015 |
| Kegler. P; Baum. G; Indriana. LF; Wild. C; Kunzmann. A | Physiological Response of the Hard Coral *Pocillopora verrucosa* from Lombok. Indonesia. to Two Common Pollutants in Combination with High Temperature | PLoS One | 10 | 2015 |
| Crook. ED; Cohen. AL; Rebolledo-Vieyra. M; Hernandez. L; Paytan. A | Reduced calcification and lack of acclimatization by coral colonies growing in areas of persistent natural acidification | PROCEEDINGS OF THE NATIONAL ACADEMY OF SCIENCES OF THE UNITED STATES OF AMERICA | 110 | 2013 |
| Hueerkamp. C; Glynn. PW; D'Croz. L; Mate. JL; Colley. SB | Bleaching and recovery of five eastern Pacific corals in an El Nino-related temperature experiment | BULLETIN OF MARINE SCIENCE | 62 | 2001 |
| Warner. ME; Fitt. WK; Schmidt. GW | The effects of elevated temperature on the photosynthetic efficiency of zooxanthellae in hospite from four different species of reef coral: A novel approach | PLANT CELL AND ENVIRONMENT | 18 | 1996 |
| Kaniewska. P; Campbell. PR; Kline. DI; Rodriguez-Lanetty. M; Miller. DJ; Dove. S; Hoegh-Guldberg. O | Major Cellular and Physiological Impacts of Ocean Acidification on a Reef Building Coral | PLOS ONE | 173 | 2012 |
| Edmunds. PJ; Cumbo. V; Fan. TY | Effects of temperature on the respiration of brooded larvae from tropical reef corals | JOURNAL OF EXPERIMENTAL BIOLOGY | 61 | 2011 |
| Comeau. S; Edmunds. PJ; Spindel. NB; Carpenter. RC | Diel pCO_2_ oscillations modulate the response of the coral *Acropora hyacinthus* to ocean acidification | MARINE ECOLOGY PROGRESS SERIES | 501 | 2014 |
| Winters. G; Beer. S; Ben Zvi. B; Brickner. I; Loya. Y | Spatial and temporal photoacclimation of *Stylophora pistillata:* zooxanthella size. pigmentation. location and clade | MARINE ECOLOGY PROGRESS SERIES | 124 | 2009 |
| Horvath. KM; Castillo. KD; Armstrong. P; Westfield. IT; Courtney. T; Ries. JB | Next-century ocean acidification and warming both reduce calcification rate. but only acidification alters skeletal morphology of reef-building coral *Siderastrea siderea* | Scientific Reports | 6 | 2016 |
| Evensen. NR; Edmunds. PJ | Conspecific aggregations mitigate the effects of ocean acidification on calcification of the coral *Pocillopora verrucosa* | JOURNAL OF EXPERIMENTAL BIOLOGY | 220 | 2017 |
| Shick. JM; Iglic. K; Wells. ML; Trick. CG; Doyle. J; Dunlap. WC | Responses to iron limitation in two colonies of *Stylophora pistillata* exposed to high temperature: Implications for coral bleaching | LIMNOLOGY AND OCEANOGRAPHY | 163 | 2011 |
| Ohki. S; Irie. T; Inoue. M; Shinmen. K; Kawahata. H; Nakamura. T; Kato. A; Nojiri. Y; Suzuki. A; Sakai. K; van Woesik. R | Calcification responses of symbiotic and aposymbiotic corals to near-future levels of ocean acidification | BIOGEOSCIENCES | 3 | 2013 |
| Camp. EF; Smith. DJ; Evenhuis. C; Enochs. I; Manzello. D; Woodcock. S; Suggett. DJ | Acclimatization to high-variance habitats does not enhance physiological tolerance of two key Caribbean corals to future temperature and pH | PROCEEDINGS OF THE ROYAL SOCIETY B-BIOLOGICAL SCIENCES | 283 | 2016 |
| Fisher. PL; Malme. MK; Dove. S | The effect of temperature stress on coral-Symbiodinium associations containing distinct symbiont types | CORAL REEFS | 97 | 2012 |
| Keshavmurthy. S; Fontana. S; Mezaki. T; Gonzalez. LD; Chen. CA | Doors are closing on early development in corals facing climate change | SCIENTIFIC REPORTS | 4 | 2014 |
| Tanaka. Y; Inoue. M; Nakamura. T; Suzuki. A; Sakai. K | Loss of zooxanthellae in a coral under high seawater temperature and nutrient enrichment | JOURNAL OF EXPERIMENTAL MARINE BIOLOGY AND ECOLOGY | 457 | 2014 |
| Lesser. MP | Oxidative stress causes coral bleaching during exposure to elevated temperatures | CORAL REEFS | 12 | 1997 |
| Cumbo. VR; Edmunds. PJ; Wall. CB; Fan. TY | Brooded coral larvae differ in their response to high temperature and elevated pCO_2_ depending on the day of release | MARINE BIOLOGY | 160 | 2013 |
| Faxneld. S; Jorgensen. TL; Tedengren. M | Effects of elevated water temperature. reduced salinity and nutrient enrichment on the metabolism of the coral *Turbinaria mesenterina* | ESTUARINE COASTAL AND SHELF SCIENCE | 188 | 2010 |
| Berkelmans. R; van Oppen. MJH | The role of zooxanthellae in the thermal tolerance of corals: a 'nugget of hope' for coral reefs in an era of climate change | PROCEEDINGS OF THE ROYAL SOCIETY B-BIOLOGICAL SCIENCES | 80 | 2006 |
| Sekizawa. A; Uechi. H; Iguchi. A; Nakamura. T; Kumagai. NH; Suzuki. A; Sakai. K; Nojiri. Y | Intraspecific variations in responses to ocean acidification in two branching coral species | MARINE POLLUTION BULLETIN | 122 | 2017 |
| Putnam. HM; Edmunds. PJ | The physiological response of reef corals to diel fluctuations in seawater temperature | JOURNAL OF EXPERIMENTAL MARINE BIOLOGY AND ECOLOGY | 130 | 2011 |
| Gibbin. EM; Putnam. HM; Gates. RD; Nitschke. MR; Davy. SK | Species-specific differences in thermal tolerance may define susceptibility to intracellular acidosis in reef corals | MARINE BIOLOGY | 162 | 2015 |
| Russell. BJ; Dierssen. HM; LaJeunesse. TC; Hoadley. KD; Warner. ME; Kemp. DW; Bateman. TG | Spectral Reflectance of Palauan Reef-Building Coral with Different Symbionts in Response to Elevated Temperature | REMOTE SENSING | 8 | 2016 |
| Goldenheim. WM; Edmunds. PJ | Effects of Flow and Temperature on Growth and Photophysiology of Scleractinian Corals in Moorea. French Polynesia | BIOLOGICAL BULLETIN | 67 | 2011 |
| Fitt. WK; Gates. RD; Hoegh-Guldberg. O; Bythell. JC; Jatkar. A; Grottoli. AG; Gomez. M; Fisher. P; Lajuenesse. TC; Pantos. O; Iglesias-Prieto. R; Franklin. DJ; Rodrigues. LJ; Torregiani. JM; van Woesik. R; Lesser. MP | Response of two species of Indo-Pacific corals. *Porites cylindrica* and *Stylophora pistillata*. to short-term thermal stress: The host does matter in determining the tolerance of corals to bleaching | JOURNAL OF EXPERIMENTAL MARINE BIOLOGY AND ECOLOGY | 121 | 2009 |
| Okazaki. RR; Towle. EK; van Hooidonk. R; Mor. C; Winter. RN; Piggot. AM; Cunning. R; Baker. AC; Klaus. JS; Swart. PK; Langdon. C | Species-specific responses to climate change and community composition determine future calcification rates of Florida Keys reefs | GLOBAL CHANGE BIOLOGY | 23 | 2017 |
| Tolosa. I; Treignier. C; Grover. R; Ferrier-Pages. C | Impact of feeding and short-term temperature stress on the content and isotopic signature of fatty acids. sterols. and alcohols in the scleractinian coral *Turbinaria reniformis* | CORAL REEFS | 93 | 2011 |
| Edmunds. PJ | Effect of elevated temperature on aerobic respiration of coral recruits | MARINE BIOLOGY | 31 | 2005 |
| Edmunds. PJ; Gates. RD; Gleason. DF | The biology of larvae from the reef coral *Porites astreoides*. and their response to temperature disturbances | MARINE BIOLOGY | 8 | 2001 |
| Comeau. S; Carpenter. RC; Lantz. CA; Edmunds. PJ | Parameterization of the response of calcification to temperature and pCO_2_ in the coral *Acropora pulchra* and the alga *Lithophyllum kotschyanum* | CORAL REEFS | 35 | 2016 |
| Gardner. SG; Raina. JB; Ralph. PJ; Petrou. K | Reactive oxygen species (ROS) and dimethylated sulphur compounds in coral explants under acute thermal stress | JOURNAL OF EXPERIMENTAL BIOLOGY | 220 | 2017 |
| Tagliafico. A; Rudd. D; Rangel. MS; Kelaher. BP; Christidis. L; Cowden. K; Scheffers. SR; Benkendorff. K | Lipid-enriched diets reduce the impacts of thermal stress in corals | MARINE ECOLOGY PROGRESS SERIES | 573 | 2017 |
| Anthony. KRN; Connolly. SR; Hoegh-Guldberg. O | Bleaching. energetics. and coral mortality risk: Effects of temperature. light. and sediment regime | LIMNOLOGY AND OCEANOGRAPHY | 159 | 2007 |
| Hoadley. KD; Pettay. DT; Grottoli. AG; Cai. WJ; Melman. TF; Levas. S; Schoepf. V; Ding. Q; Yuan. XC; Wang. YC; Matsui. Y; Baumann. JH; Warner. ME | High-temperature acclimation strategies within the thermally tolerant endosymbiont *Symbiodinium trenchii* and its coral host. *Turbinaria reniformis*. differ with changing pCO_2_ and nutrients | MARINE BIOLOGY | 163 | 2016 |
| Buxton. L; Takahashi. S; Hill. R; Ralph. PJ | Variability in the primary site of photosynthetic damage in *Symbiodinium* sp. (Dinophyceae) exposed to thermal stress. | JOURNAL OF PHYCOLOGY | 152 | 2012 |
| Putnam. HM; Davidson. JM; Gates. RD | Ocean acidification influences host DNA methylation and phenotypic plasticity in environmentally susceptible corals | EVOLUTIONARY APPLICATIONS | 9 | 2016 |
| Beraud. E; Gevaert. F; Rottier. C; Ferrier-Pages. C | The response of the scleractinian coral *Turbinaria reniformis* to thermal stress depends on the nitrogen status of the coral holobiont | JOURNAL OF EXPERIMENTAL BIOLOGY | 216 | 2013 |
| COLES. SL; JOKIEL. PL | Synergistic effects of temperature, salinity, and light on hermatypic coral *Montipora verrucosa.* | MARINE BIOLOGY | 52 | 1978 |
| Nystrom. M; Nordemar. I; Tedengren. M | Simultaneous and sequential stress from increased temperature and copper on the metabolism of the hermatypic coral *Porites cylindrica* | MARINE BIOLOGY | 7 | 2001 |

Anton, A., Randle, J. L., Garcia, F. C., Rossbach, S., Ellis, J. I., Weinzierl, M., & Duarte, C. M. (2020). Differential thermal tolerance between algae and corals may trigger the proliferation of algae in coral reefs. *Global Change Biology*.

Arrhenius, S. (1889). Über die Dissociationswärme und den Einfluss der Temperatur auf den Dissociationsgrad der Elektrolyte. *Zeitschrift für physikalische Chemie, 4*(1), 96-116.

Brown, J. H., Gillooly, J. F., Allen, A. P., Savage, V. M., & West, G. B. (2004). Toward a metabolic theory of ecology. *Ecology, 85*(7), 1771-1789.

Canavero, A., Arim, M., Pérez, F., Jaksic, F. M., & Marquet, P. A. (2018). A metabolic view of amphibian local community structure: the role of activation energy. *Ecography, 41*(2), 388-400. doi:10.1111/ecog.02336

Chan, N., & Connolly, S. R. (2013). Sensitivity of coral calcification to ocean acidification: a meta‐analysis. *Global Change Biology, 19*(1), 282-290.

Clausen, C. (1971). *Effects of temperature on the rate of 45 calcium uptake by Pocillopora damicornis*: University of Hawaii Press, Honolulu.

Gibert, J. P., Chelini, M.-C., Rosenthal, M. F., & DeLong, J. P. (2016). Crossing regimes of temperature dependence in animal movement. *Global Change Biology, 22*(5), 1722-1736. doi:10.1111/gcb.13245

Gillooly, J. F., Brown, J. H., West, G. B., Savage, V. M., & Charnov, E. L. (2001). Effects of size and temperature on metabolic rate. *science, 293*(5538), 2248-2251.

Hedges, L. V., Gurevitch, J., & Curtis, P. S. (1999). The meta‐analysis of response ratios in experimental ecology. *Ecology, 80*(4), 1150-1156.

Kordas, R. L., Harley, C. D., & O'Connor, M. I. (2011). Community ecology in a warming world: the influence of temperature on interspecific interactions in marine systems. *Journal of Experimental Marine Biology and Ecology, 400*(1-2), 218-226.

Savva, I., Bennett, S., Roca, G., Jordà, G., & Marbà, N. (2018). Thermal tolerance of Mediterranean marine macrophytes: Vulnerability to global warming. *Ecology and Evolution, 8*(23), 12032-12043. doi:10.1002/ece3.4663

Wang, J.-T., Meng, P.-J., Chen, Y.-Y., & Chen, C. A. (2012). Determination of the thermal tolerance of Symbiodinium using the activation energy for inhibiting photosystem II activity. *Zoological Studies, 51*(2), 137-142.

Wang, J.-T., Wang, Y.-T., Keshavmurthy, S., Meng, P.-J., & Chen, C. A. (2019). The coral Platygyra verweyi exhibits local adaptation to long-term thermal stress through host-specific physiological and enzymatic response. *Scientific reports, 9*(1), 1-11.

Weber, J., & White, E. (1974). Activation energy for skeletal aragonite deposited by the hermatypic coral Platygyra spp. *Marine Biology, 26*(4), 353-359.

Wittmann, A. C., & Pörtner, H.-O. (2013). Sensitivities of extant animal taxa to ocean acidification. *Nature Climate Change, 3*(11), 995.
